# Efficiency of single-pulse laser fragmentation of organic nutraceutical dispersions in a circular jet flow-through reactor

**DOI:** 10.3762/bjnano.16.55

**Published:** 2025-05-26

**Authors:** Tina Friedenauer, Maximilian Spellauge, Alexander Sommereyns, Verena Labenski, Tuba Esatbeyoglu, Christoph Rehbock, Heinz P Huber, Stephan Barcikowski

**Affiliations:** 1 Technical Chemistry I and Center for Nanointegration Duisburg-Essen (CENIDE), University of Duisburg-Essen, Universitaetsstrasse 7, Essen 45141, Germany; 2 Department of Applied Sciences and Mechatronics, Lasercenter HM, Munich University of Applied Sciences HM, Lothstrasse 34, Munich 80335, Germany; 3 Institute of Photonic Technologies (LPT), Friedrich-Alexander-Universität Erlangen-Nürnberg, Konrad-Zuse-Strasse 3/5, Erlangen 91052, Germany; 4 Erlangen Graduate School in Advanced Optical Technologies (SAOT), Friedrich-Alexander-Universität Erlangen-Nürnberg, Paul-Gordan-Strasse 6, Erlangen 91052, Germany; 5 Department of Molecular Food Chemistry and Food Development, Institute of Food and One Health, Gottfried Wilhelm Leibniz University Hannover, Am Kleinen Felde 30, Hannover 30167, Germany

**Keywords:** antioxidant, cannabidiol, curcumin, drug, food additive, low degradation, nanoparticle, pulsed laser ablation in liquids, solubilization

## Abstract

Nutraceuticals provide health benefits and particularly profit from a sensitive, high-purity production process. Microparticle laser fragmentation in liquids is an emerging technique for the contamination-free comminution of organic drugs and nutraceuticals aiming at solubility enhancements. However, current discontinuously operated fragmentation setups suffer from chemical degradation by multipulse laser excitation at high fluence and do not allow for systematic studies of the fragmentation mechanisms. In this work, continuous-flow microparticle laser fragmentation in liquids with ultrashort-pulsed lasers was studied in a circular jet reactor using curcumin and cannabidiol as model substances and single-pulse-per-volume element conditions to compare the fragmentation efficiency for these two nutraceuticals. Fragmentation efficiency based on the yield of submicrometer particles and nanoparticles was quantified using UV–vis extinction spectroscopy, scanning electron microscopy, and analytical centrifugation, while high-performance liquid chromatography determined degradation. We found improved fragmentation efficiency at lower mass concentrations. In all experiments, chemical degradation was minimal (<2%), and increased mass concentration of curcumin enabled ultralow by-product formation of 0.01%. The process selectivity against degradation was defined by the application-relevant descriptor of mole degradation per produced submicrometer particle surface and quantified regarding feedstock mass concentration and nutraceutical type. Cytotoxicity in HepG2 cancer cells was significantly reduced in cells treated with laser-processed curcumin in comparison to unirradiated curcumin controls, and antioxidant effects were proven, ensuring high viability even at high curcumin concentrations.

## Introduction

Laser synthesis and processing of colloids (LSPC) has become increasingly popular over the last few decades, as this relatively new fabrication method can be used to produce stable, additive-free colloids of different material classes under high-purity conditions, which are suitable for a wide range of technical applications [1–5]. Pulsed laser ablation (LAL), laser fragmentation (LFL), and laser melting (LML) in liquids are aimed at synthesizing nanoparticles (NPs) from bulk targets (LAL), by downsizing (LFL), or by increasing/reshaping (LML) particle dispersions [1]. On the other hand, pulsed laser defect engineering in liquids (PUDEL) processes involve targeted post-treatment of colloids, for example, to increase their defect density in favor of electrocatalytic or optical properties without changing their size [6–8]. In addition, the acronym PUDEL has recently been used for pulsed laser diffusion enhancement in liquids, which refers to an increase in diffusion without influencing the particle size or morphology [9], working at comparably low laser fluences (PUDEL < LML < LAL ≤ LFL). Recently, the pulsed laser extraction of organic matter in liquid (LEL), in the mild fluence regime of PUDEL, has been demonstrated to be much faster and more efficient than state-of-the-art extraction methods, exemplified by extraction from coffee powders [10] and alkaloid drug extraction from ground root powder [11]. Initially, LSPC research focused on inorganic materials such as metals [12–13], semiconductors [1], and oxides [14–15], where the particle formation mechanisms are well understood. In the last two decades, the transfer of these laser-based processes to organic substances has been reported with a particular focus on particle size reduction by LFL [10,16–17]. One motivation behind the size reduction of organic particles like drugs or near-infrared absorbing dyes, which often are hydrophobic, is their increased solubilization or aqueous dispersion, relevant for pharmacological, photochemical, and photomedical applications. For example, metal complex phthalocyanines (Pc) [18] such as vanadium (VOPc) [19–22], copper (CuPc) [23–24], aluminum (AlPc) [24], and iron phthalocyanines (FePc), but also some purely organic dyes such as naphthalocyanides [25], perylenes [26], perylene diimides [27–28], fullerenes [29–30], and quinacridones [2,31] have been successfully downsized to the nanoscale using nanosecond- and femtosecond-LFL with wavelengths in the UV, green, and IR ranges. Metal complex dyes are characterized by high thermal and UV stability and were therefore preferred for initial LFL studies [32]. Asahi et al. and Tamaki et al. repeatedly showed that using a UV nanosecond laser with a repetition rate of several hertz not only produces NPs of phthalocyanines (VOPc and CuPc) in deionized water and other pure solvents but also influences the size and crystal phase via the chosen laser parameters [21,33]. Furthermore, spectroscopic analyses successfully demonstrated that an extinction enhancement occurs with increasing irradiation time and higher fluences, which could be explained by NP formation [22,34]. However, this increase in extinction could only be observed once a certain threshold fluence was exceeded. If the laser fluence is further increased above a critical value, degradation can occur due to excessive energy input. Using fullerene C_60_ as an example, Sugiyama et al. showed that an increase in fluence from 50 to 100 mJ·cm^−2^ using a UV laser at 355 nm leads to an extinction enhancement and thus an increase in the number of NPs. However, when a value of 150 mJ·cm^−2^ is exceeded, the laser-generated NPs appear colorless, indicating chemical degradation [29]. Other purely organic materials, such as vitamin C and capsaicin (an alkaloid in chili), were also fragmented using a femtosecond laser with a wavelength of 800 nm, and the yield was determined based on an increase in extinction using UV–vis extinction spectroscopy. It was shown that a 36% increase in the specific extinction band could be achieved for vitamin C and up to 135% for capsaicin [35]. Further work demonstrated the successful laser-induced particle size reduction of curcumin, where the laser-generated NPs had particle sizes below 500 nm. These NPs also exhibited greater intracellular uptake and increased cytotoxicity on rat C6 glioma cells [36]. Laser-generated nanoscale cinnamon was also synthesized and showed enhanced antibacterial activity against Gram-negative and Gram-positive bacteria after particle size reduction compared to the unirradiated educt [37].

**Figure 1:**
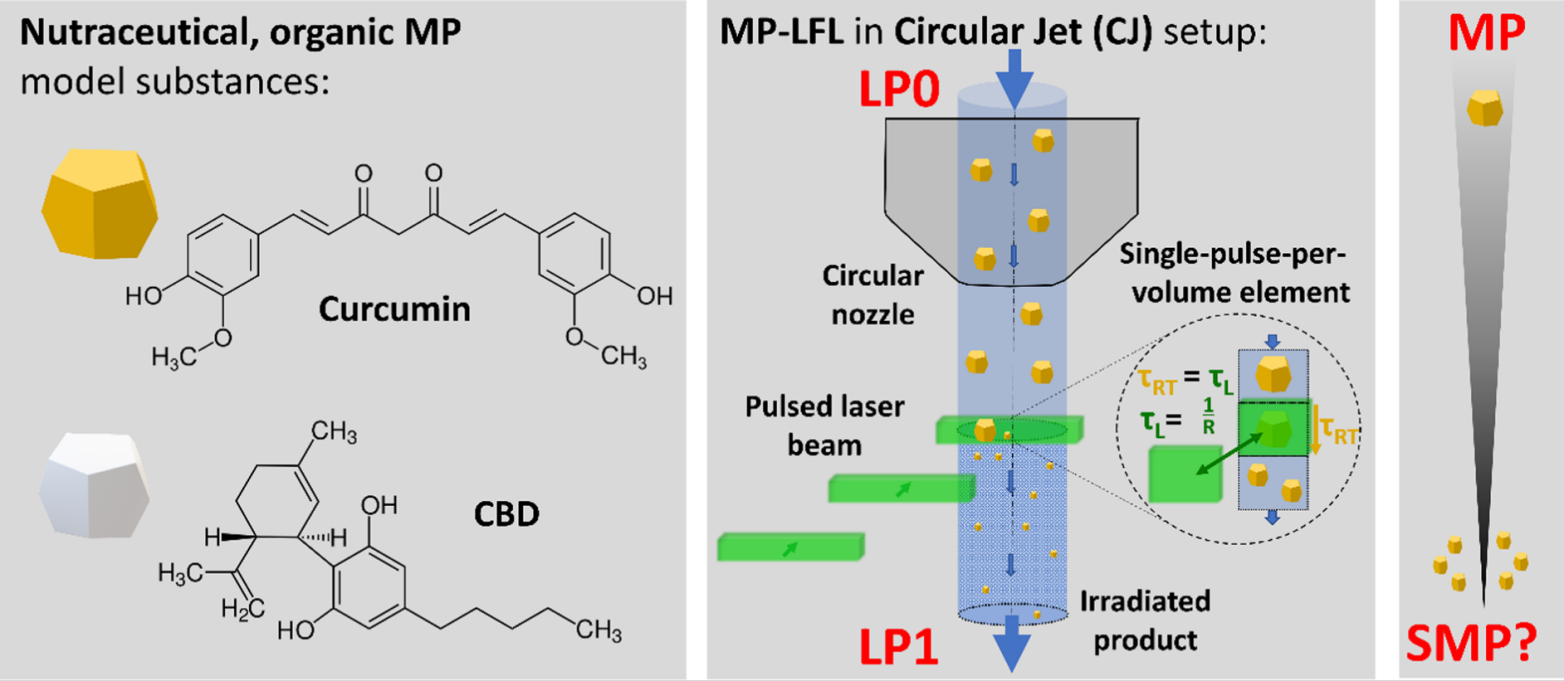
Nutraceutical model substances curcumin and cannabidiol (CBD) and circular jet (CJ) flow-through fragmentation setup under single-pulse-per-volume conditions, downsizing the microparticle (MP) feedstock dispersion into submicrometer particles (SMPs). Single-pulse conditions are achieved if the residence time of the volume flow τ_RT_ is equal to the laser pulse interval τ_L_ (reciprocal of the repetition rate R).

The LFL of lipophilic drugs is of particular interest in improving their dissolution properties and oral absorption. Better dissolution properties and dissolution rates lead to a higher saturation limit, which allows one to reduce the required amount of the drug [38]. However, for sensitive active pharmaceutical ingredients (APIs), a contamination-free and degradation-free manufacturing process must be ensured, which requires fine-tuning of the laser parameters. Meunier et al. reported that successful NP generation of megestrol acetate [39], paclitaxel [40], beclomethasone dipropionate [41], naproxen, and fenofibrate [42] by LFL is possible; however, depending on the applied power and irradiation time, an inversely proportional relationship between particle size and the portion of degradation products was reported. Previous studies using naproxen and fenofibrate as model drugs showed that using nanosecond lasers with a wavelength of 532 nm resulted in more degradation products compared to lasers with femtosecond pulse duration and a wavelength in the IR range [42]. Furthermore, using beclomethasone dipropionate as an example, it was also demonstrated that no submicrometer particles (SMPs) <1000 nm can be generated using nanosecond IR lasers [41]. A direct size comparison of naproxen and fenofibrate particles generated by nanosecond and femtosecond lasers showed that shorter pulse durations favor the generation of smaller NPs, analogous to the dye studies described above. However, in addition to laser parameters such as wavelength and pulse duration, fragmentation setups can also affect the fragmentation efficiency. A homogeneous fluence distribution is particularly relevant for sensitive organic materials, as the amount of degradation products scales with the laser intensity used [42], which is why fluence gradients and particularly high peak intensities are undesirable and should be minimized to produce high-quality and uniform organic SMPs and NPs using LSPC. All LFL experiments described and discussed above were performed in a closed batch setup. These setups consist of glass vessels with stirrers containing the NP or microparticle (MP) dispersions, which are irradiated with a laser beam. However, as dispersion volumes in batch setups are only partially illuminated and there is constant back-diffusion, no uniform fluence and number of pulses-per-particle can be guaranteed. This prohibits systematic studies of the fragmentation mechanism and reliable measures of fluence-dependent degradation and particle size. To compensate for this disadvantage, Lau et al. introduced a circular jet (CJ) flow-through reactor [15], which generates a hydrostatically driven free liquid jet that is perpendicularly irradiated by a focused laser beam (Figure 1). By matching the flow rate of the dispersion in the jet, which defines the residence time of the particles in the laser-excited volume, with the repetition rate of the laser, precise process control over the pulses per particle is possible [43]. Hence, the use of the CJ allows for a more uniform fluence distribution and better control over the number of pulses-per-volume element (PPV) than batch setups. For example, Friedenauer et al. described the superior fragmentation efficiency in a CJ compared to the batch setup using the model drug naproxen. CJ picosecond-LFL at a wavelength of 515 nm resulted in a 100 times higher yield of SMPs <1000 nm at the same number of applied laser pulses [43]. However, despite the superior fragmentation efficiency in the CJ compared to batch setups, the lowest PPV value tested was 20, and the laser processing steps (passages) needed to be repeated five times to achieve maximal conversion of MPs into SMPs. Moreover, single-PPV (equal to single-pulse-per-particle) conditions allow for a better understanding of the role of feedstock particle size, concentration, and optical extinction without the particle properties being transiently affected by multiple laser pulses. Here, nutraceuticals, defined as plant-based foods with scientifically approved health-related effects [44–45], are a good model material as they, on the one hand, require sensitive processing methods and, on the other hand, are highly application-relevant at the interface between nutritional science and the pharmaceutical industry [46–47].

This study aims to elucidate how an organic MP suspension needs to be designed to enable energy- and process-efficient fragmentation with just one nominal laser pulse-per-particle (and practically only one passage of the jet passing the laser) in a liquid jet (Figure 1). The two organic model nutraceuticals curcumin and cannabidiol (CBD) were used to cover different material classes with different initial particle diameters. As triggering photomechanical fragmentation effects is intended [43], we used an ultrashort-pulsed laser. We choose picosecond pulse duration to avoid the risk of optical breakdown in the liquid at shorter pulse durations [48]. In addition to the comminution efficiency, the formation of degradation products was quantitatively evaluated as a high degree of product purity is required, especially when processing sensitive nutraceuticals intended for the use as food additives or medical applications. This is complemented by evaluations of the biocompatibility and antioxidant features (i.e., suppression of reactive oxygen species (ROS)) of laser-processed curcumin.

## Results and Discussion

**Figure 2:**
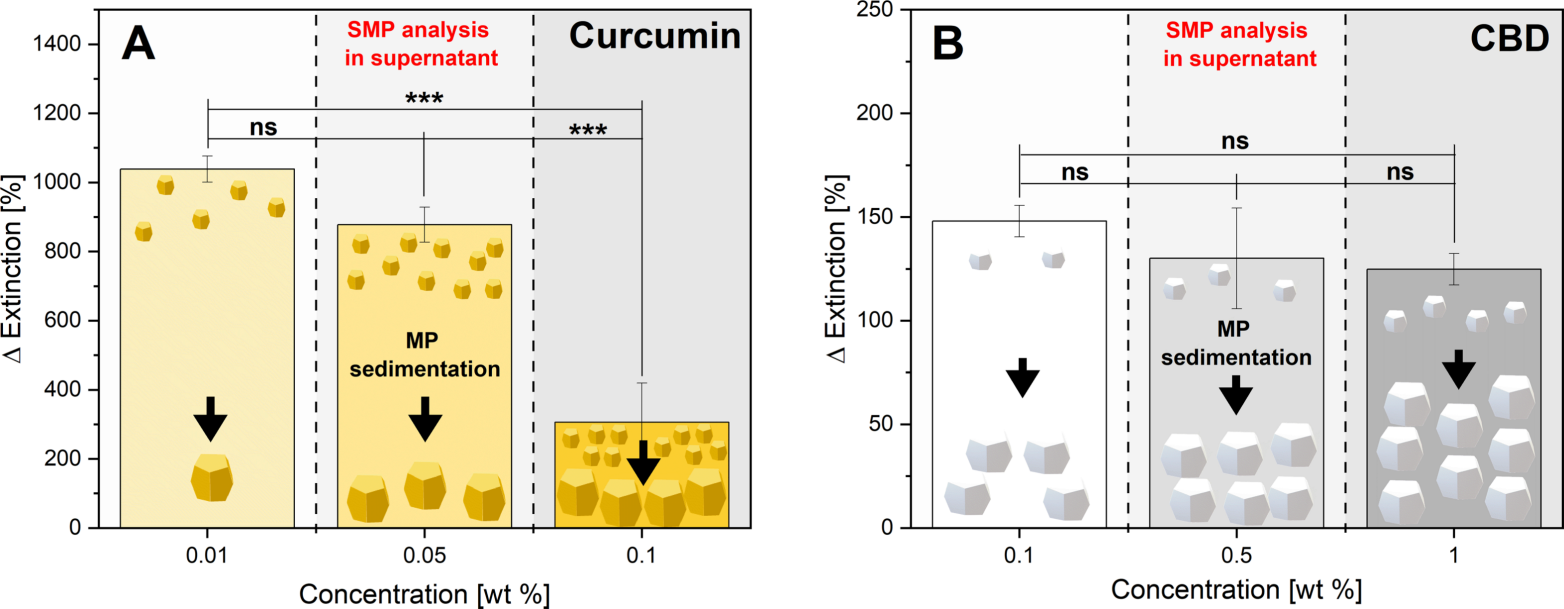
Impact of mass concentration effects on extinction enhancement determined via UV–vis extinction spectroscopy for curcumin (A) and CBD (B) after MP-LFL (from LP0 to LP1) in the CJ setup at a fluence of (284 ± 45) mJ·cm^−2^ and under near single-pulse conditions. The bar charts show the mean values of three independent measurements (*n* = 3). The error bars indicate the standard deviation. Asterisks indicate the significance of differences between the concentration-dependent extinction enhancement determined by one-way analysis of variance (1-way ANOVA) with a significance level α of 0.05. Values of *P* ≤ 0.05 are summarized with one star, values of *P* ≤ 0.01 are summarized with two stars, and values of *P* ≤ 0.001 are given with three stars. Values of *P* > 0.05 were marked as not significant (“ns” in the graphs). The scheme illustrates the two relevant particle size fractions with non-fragmented educt MPs being removed by sedimentation (depicted by the arrows) before the measurements, and smaller particles (NPs and SMPs) retained and quantified in the supernatant. The different numbers of large and small particles in the cartoon clarify that ΔExtinction is a relative and not a total increase in supernatant extinction.

**Figure 3:**
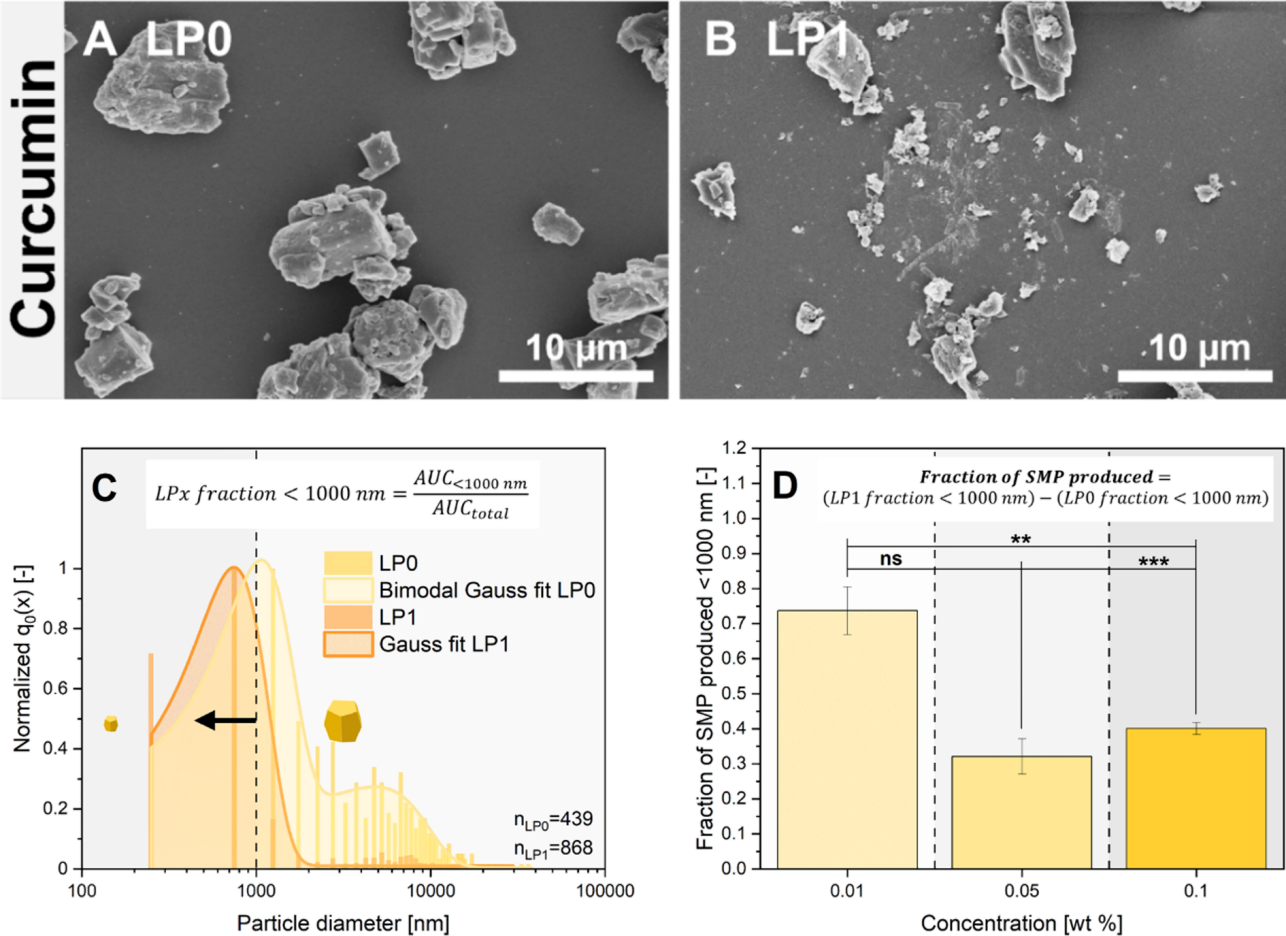
Particle size and shape of curcumin. SEM image of the educt material (A) and the fragmented particles (B). Exemplary number-weighted particle diameter distributions of irradiated (LP1) and non-irradiated (LP0) samples (C) determined via SEM and ImageJ (0.1 wt %). The equation in the insert indicates how fractions of particles <1000 nm (SMPs) were calculated from these distributions by using the area under the curve (AUC) of the corresponding particle size distributions. The fraction of SMPs produced via LFL (D) and how it depends on educt mass concentration. The equation in the insert shows how the number of produced SMPs was calculated by subtracting the number of SMPs in the educt dispersions (LP0) from those found after LP1. The error bars show the standard deviation of three independent measurements. Asterisks indicate the significance of differences between the concentration-dependent SMP production determined by one-way analysis of variance (1-way ANOVA) with a significance level α of 0.05. Values of *P* ≤ 0.05 are summarized with one star, values of *P* ≤ 0.01 are summarized with two stars, and values of *P* ≤ 0.001 are given with three stars. Values of *P* > 0.05 were marked as not significant (“ns” in the graphs).

**Figure 4:**
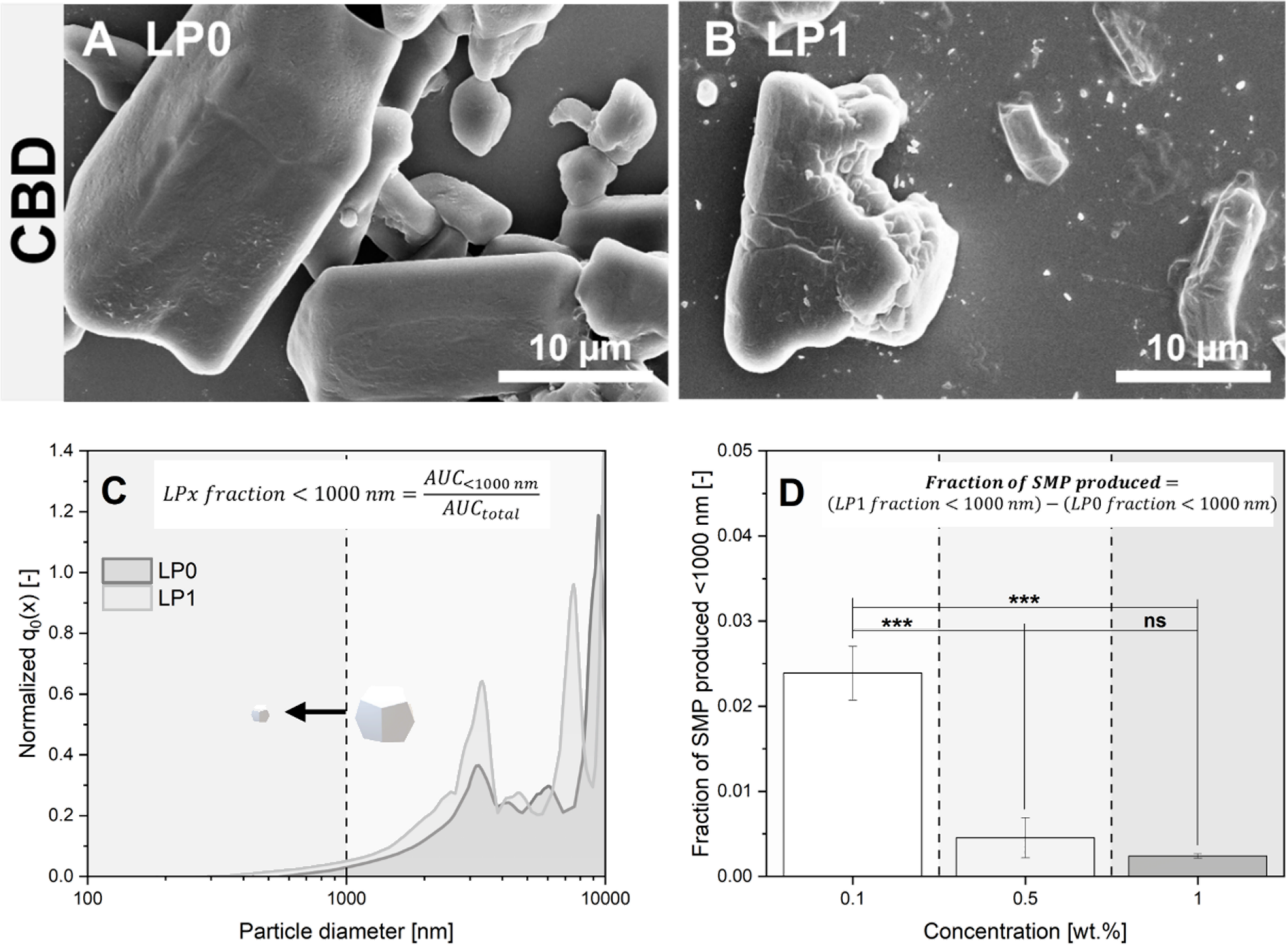
Particle size and shape of CBD. SEM image of the educt material (A) and the fragmented particles (B). Exemplary number-weighted particle diameter distributions of irradiated (LP1) and non-irradiated (LP0) samples (C) determined via SEM and ImageJ (0.1 wt %). The equation in the insert indicates how fractions of particles <1000 nm were calculated from these distributions by using the area under the curve (AUC) of the corresponding particle size distributions. The fraction of SMPs (<1000 nm) produced via LFL (D) and how it depends on educt mass concentration. The equation in the insert shows how the number of produced SMPs was calculated by subtracting the number of SMPs in the educt dispersions (LP0) from those found after LP1. Error bars show the standard deviation of three independent measurements. Asterisks indicate the significance of differences between the concentration-dependent SMP production determined by one-way analysis of variance (1-way ANOVA) with a significance level α of 0.05. Values of *P* ≤ 0.05 are summarized with one star, values of *P* ≤ 0.01 are summarized with two stars, and values of *P* ≤ 0.001 are given with three stars. Values of *P* > 0.05 were marked as not significant (“ns” in the graphs).

To evaluate the laser fragmentation efficiency in the CJ setup, the nutraceuticals curcumin and CBD (Figure 1) were dispersed at different concentrations (0.01 to 1.0 wt %) in deionized water and homogenized by ultrasonication. The MP-LFL yield of SMPs and NPs was quantified by the extinction enhancement in the colloidally stable supernatants of particle dispersions after sedimentation of the colloidally unstable, unfragmented MP educts, determined by UV–vis extinction spectroscopy, which is proportional to the amount of SMPs and NPs [2,22,49]. This extinction enhancement from the unirradiated sample (no laser passage, LP0) to one laser passage (LP1, equal to nominal 1 PPV) for both substances, curcumin and CBD, is depicted in Figure 2. Furthermore, the supernatants of the samples, which contained SMPs and NPs, were mixed with acetonitrile and analyzed by UV–vis extinction spectroscopy (Figures S1 and S2, Supporting Information File 1). It is observable, that at low concentrations (0.01 wt % for curcumin and 0.1 wt % for CBD), the relative increase in extinction is more pronounced than at high concentrations. At a curcumin concentration of 0.01 wt %, the increase in extinction from LP0 to LP1 is 1038%. At 0.05 wt % it is still 878%, whereas at 0.1 wt % only 306% is achieved (absolute values in Figure S1A and Figure S2A, Supporting Information File 1). This relative boost in extinction at low concentrations can be seen with both model substances, although a statistically significant distinction is only possible for the LFL of curcumin. It should be noted that ten times lower mass concentrations were used for curcumin, as this feedstock material has about a ten times smaller size (see below in Figure 3 and Figure 4) and therefore already has a high number concentration. As expected, an increase in the mass concentration of the feedstock MPs leads to a reduction in the mass-specific fragmentation efficiency for both model substances due to the effect of laser fluence attenuation based on the Beer–Lambert law in the beam path penetrating the liquid jet.

Motivated by previous studies on organic drugs such as paclitaxel [40] and fenofibrate [42] and on C_60_ and VOPc [2,22,34], where a reduction of particle size distributions with increasing laser fluence was reported (at either undefined or high PPV values), we also analyzed the particle size distribution of the educt and product particles of curcumin and CBD evaluating the fraction of produced SMPs and NPs after LP1.

The previously discussed higher UV–vis extinction in the supernatants of the LFL-treated samples is already a good indicator of a shift of the particle size distributions towards smaller particles (as extinction increases with particle number concentration caused by downsizing). These findings were confirmed by complementary assays, directly comparing particle diameter distributions of the single-pulse-irradiated (LP1) and non-irradiated (LP0) samples. As marginal particle size changes also needed to be considered, the number-weighted particle density distribution was used. The corresponding results are shown in Figure 3 for curcumin and in Figure 4 for CBD. The exemplary SEM images of curcumin in Figure 3A of the starting material and Figure 3B of the laser-treated product qualitatively indicate a recognizable reduction in particle diameters. Moreover, pronounced morphological changes caused by thermal effects (i.e., LML) like spheroidization of the MPs cannot be observed. This points towards the occurrence of laser fragmentation already after one laser processing step under near single-pulse conditions. However, even after fragmentation, several larger particles with irregular and angular shapes, as well as some large educt particles, seemingly not affected in size by the laser, are still observed besides many small fragments. Please note that the presence of very small NPs (2–3 nm), as observed in MP-LFL experiments of IrO_2_ [14], is not ruled out, but these cannot be detected with the measurement methods used for organic materials. The number-weighted particle size distribution determined via SEM in Figure 3C at the example of 0.1 wt % curcumin shows that the initially polydisperse, bimodal distribution with modal values of *x*_mode 1, LP0_ = (1.04 ± 0.05) µm and *x*_mode 2, LP0_ = (5.06 ± 0.29) µm is transformed into a monomodal distribution with *x*_mode, LP1_ = (0.70 ± 0.07) µm. Modal values for all concentrations are listed in Table S3, Supporting Information File 1. As the number-weighted distribution *q*_0_(*x*) is shown here, fractions of larger educt particles are negligible in number in the distribution curve after fragmentation. To evaluate the fragmentation efficiency, as previously described by Friedenauer et al. [43], the area under the curve (AUC) of the size histogram with a cut-off < 1000 nm and the total AUC with a cut-off < 10000 nm were calculated by integration, in each case for LP0 (= LP0 fraction < 1000 nm) and LP1 (= LP1 fraction < 1000 nm), to determine the portion of particles <1000 nm (equation inset Figure 3C). However, the unfragmented LP0 samples may already contain a low number-fraction of particles <1000 nm. To account for this, the difference between the values of the LP1 and LP0 fractions <1000 nm is used to calculate a normalized value of the curcumin SMPs produced, representing the fragmentation efficiency (equation inset Figure 3D). Analogously to the previously discussed increase in the optical extinction of the curcumin dispersion, it can be seen that the particle size reduction is significantly more effective at low concentrations than at high concentrations. Best size reduction effects were observed at 0.01 wt % curcumin validating the trends derived from the optical supernatant characterization. Analogously to Figure 3, Figure 4A and Figure 4B show, the CBD educt and the corresponding fragmented sample after LP1, respectively. The SEM images post-LFL indicate the presence of SMPs <1000 nm, which were not found in the educt samples. Since the CBD educt particles are one order of magnitude larger compared to the curcumin particles, which is also reflected in the number-weighted particle density distribution in Figure 4C, and the density of both materials is similar, the method of analytical centrifugation is useful to compare the hydrodynamic particle size distribution, providing better statistics than SEM. It should be noted here that the analytical centrifuge can, at the given density of organic particles, quantitatively characterize particle suspensions in the range of 100–10000 nm, depending on the used sedimentation protocols; size fractions outside this range cannot be reliably quantified (which is why we additionally determined a particle size distribution based on SEM images, Figure S6, Supporting Information File 1). The comminution of CBD by MP-LFL is also recognizable, but only few particles are <1000 nm. Accordingly, the CBD content of particles <1000 nm only reaches low values for the fraction of SMPs produced via LFL, which is also consistent with the extinction enhancement results from Figure 2B. Figure 4D also shows that low mass concentrations in the CJ setup result in the most efficient particle size reduction. Based on the combined fragmentation efficiency determined by two complementary methods, we can conclude that the concentration dependency of the fragmentation efficiency is very pronounced with a high efficiency at lower mass concentrations and a pronounced efficiency dip at higher concentrations.

**Figure 5:**
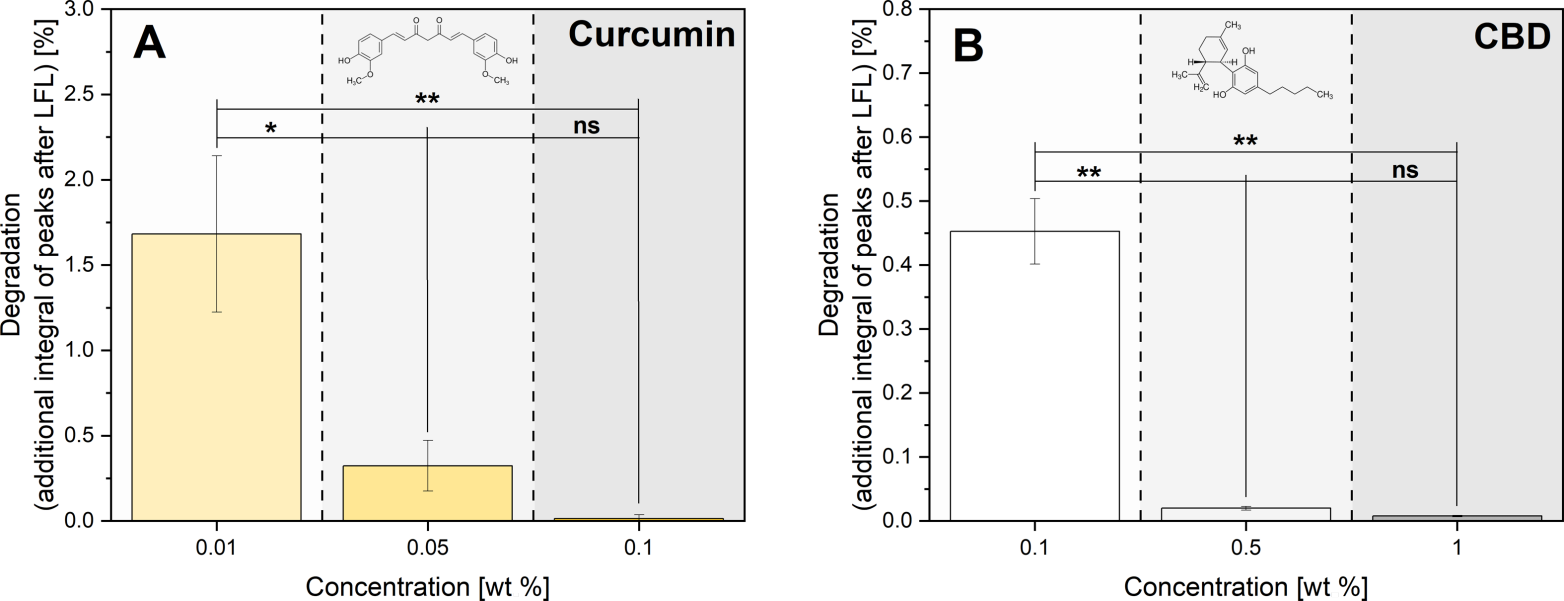
Impact of nutraceutical feedstock MP concentration on the amount of degradation products determined by RP-HPLC measurement for curcumin (A) and CBD (B) after single-pulse MP-LFL in the liquid jet. The bar charts show the mean values of three independent measurements (*n* = 3) with the standard deviation as the error bars. Asterisks indicate the significance of differences between the concentration-dependent amount of degradation determined by one-way analysis of variance (1-way ANOVA) with a significance level α of 0.05. Values of *P* ≤ 0.05 are summarized with one star, values of *P* ≤ 0.01 are summarized with two stars, and values of *P* ≤ 0.001 are given with three stars. Values of *P* > 0.05 were marked as not significant (“ns” in the graphs).

Since the portion of degradation products, which characterizes the chemical stability of nutraceuticals against LFL, is also relevant to evaluate the efficiency of the fragmentation process, impurities after fragmentation were quantified using HPLC analysis. Moreover, quantifying degradation is important for product application-oriented statements. All newly appearing peaks were assumed to be LFL-caused impurities (Figures S7–S12, Supporting Information File 1). The portions of additional peaks appearing (Figure 5A for curcumin and Figure 5B for CBD) are highest with 1.68% for low curcumin concentrations of 0.01 wt % after LP1, which is in good agreement with the fragmentation efficiency results showing the highest extinction enhancement of more than 1000% (Figure 2A). Hence, high fragmentation efficiency goes along with an elevated portion of degradation products. In contrast, at higher particle mass concentrations in the dispersion, relatively fewer particles are fragmented because of shielding effects, which accordingly leads to lower quantities of degradation products. Corresponding to the lower increase in extinction at 0.05 wt % (878%), the quantities of degradation products are also lower, and degradation products at a portion of 0.32% are found. A further increase in concentration to 0.1 wt % (with an extinction enhancement of 306%) leads to 0.01% degradation for curcumin. Interestingly, also the absolute values of chemical degradation are reduced by a factor of ten when the mass concentration of curcumin increases tenfold. The degradation of curcumin can occur either by thermal or by photochemical channels. Under the conditions examined herein, thermal degradation is unlikely to occur to a large extent as curcumin has a comparatively high melting temperature of 456–459 K [50], which is not permanently exceeded when ultrashort picosecond laser pulses are used (calculated temperatures of the particle ensemble and individual particles are listed in Table S11, the phase diagram is depicted in Figure S16, Supporting Information File 1) [51–52]. For curcumin (and other organic materials), degradation already takes place in the melting temperature range, which is why the melting temperature roughly corresponds to the degradation temperature [53–56]. Another potential mechanism would be photochemical degradation. It has been reported that upon irradiation the curcumin molecule dissociates primarily at its central diketone moiety, initiating the formation of vanillin and ferulic acid [57–58]. This is in line with the formation of lower molecular weight products indicated in Figures S7–S9, Supporting Information File 1. Furthermore, polymerization of these phenolic compounds is reported [58], which is verified by small peaks at higher retention times in the HPL chromatograms. The photochemical degradation could occur via two partially interacting pathways, namely, (I) direct damage by the particle fragmentation process or (II) indirect damage by ROS resulting either from the optical breakdown of the solvent or by-products from the direct mechanism. The contributions from the direct damage pathway are verified by the fact that degradation and particle fragmentation efficiency are proportional (Figure 3, Figure 5A). However, the pronounced concentration dependency with a ten times higher absolute molecular degradation when the concentration is reduced by a factor of ten (0.1 wt % versus 0.01 wt %) warrants further explanations. One main contribution in this context is optical shielding following the Beer–Lambert (or Bouguer–Lambert) law. Furthermore, laser-induced optical breakdown effects causing the production of ROS are also conceivable. Even though the laser intensities applied at the jet’s entrance (ca. 3·10^10^ W·cm^−2^) are one order of magnitude below the irradiance threshold range for the optical breakdown of water (i.e., 4.5·10^11^ W·cm^−2^ at 30 ps, 1064 nm [59]), it is difficult to pinpoint to which extent such nonlinear, free-electron-generating, radical-promoting effects need to be considered as a contribution to the degradation of the nutraceuticals in our specific setup. On the one hand, it is well-known that the threshold of the Kerr effect is considerably lower in the presence of (inorganic) nanoparticles [60]. On the other hand, it has been shown that optical breakdown and the production of ROS are hampered at high nanoparticle concentrations [60], which may be responsible for the low degradation by LFL-generated radicals at high particle mass concentrations. Furthermore, it has to be considered that curcumin is not only susceptible to photodegradation but is also a known radical scavenger [61–62]. These two opposing mechanisms, which most likely have a different concentration dependency, may also be responsible for the higher chemical degradation at low concentrations. It has been reported that curcumin mostly functions as a radical scavenger at higher concentrations [63]. Further studies also report the photodegradation kinetics of phenolic compounds with antimicrobial properties to be inversely dependent on concentration [64].

For CBD with the same mass concentration of 0.1 wt % of active ingredient, a relative extinction enhancement of only 148% is achieved, which corresponds to less than half the extinction enhancement of curcumin, however, with 0.45% degradation products (ca. 40 times higher than in curcumin). Consequently, the LFL of curcumin occurs not only with a higher efficiency (higher yield of small particles) but also with a lower chemical degradation in comparison to CBD. This means curcumin LFL has a higher selectivity (produced SMPs versus degradation by-products). Overall, there is a material-dependent difference in the fragmentation process of the two model materials, with curcumin not only reaching higher yields but also higher product selectivity.

Nevertheless, it is noticeable as a common trend for both model substances that low concentrations (0.01 wt % curcumin or 0.1 wt % CBD) lead to the best increase in fragmentation efficiency, but also result in an increased formation of degradation products, though the total level of degradation is still relatively low. Conventional comminution methods for organic materials such as wet or cryogenic grinding commonly yield 6% to 8% of degradation products when reaching SMP and NP sizes [65–66]. This is by a factor of 2–4 higher compared even to the highest degradation value (<2%) found in the present study on nutraceutical comminution by a picosecond-pulsed laser. LFL studies for organic drugs such as fenofibrate and naproxen have shown that LFL offers a high potential for particle size reduction of organic materials, but because of the inefficient irradiation in the batch setup and the constant back-diffusion, a high number of pulses is required, resulting in up to 23% degradation products once SMPs are generated [42]. In our previous work, we have also shown that a 100 times higher fragmentation efficiency of the model substance naproxen is achieved when using a CJ reactor instead of batch setups. By forming a thin liquid jet and controlling the PPV, the number of pulses required for a given yield was significantly reduced, which also led to a reduction of degradation products. After the complete transformation of all MPs into SMPs (yield of 100%), less than 1% degradation products were detected in naproxen [43], which is in good agreement with the findings for curcumin and CBD.

**Figure 6:**
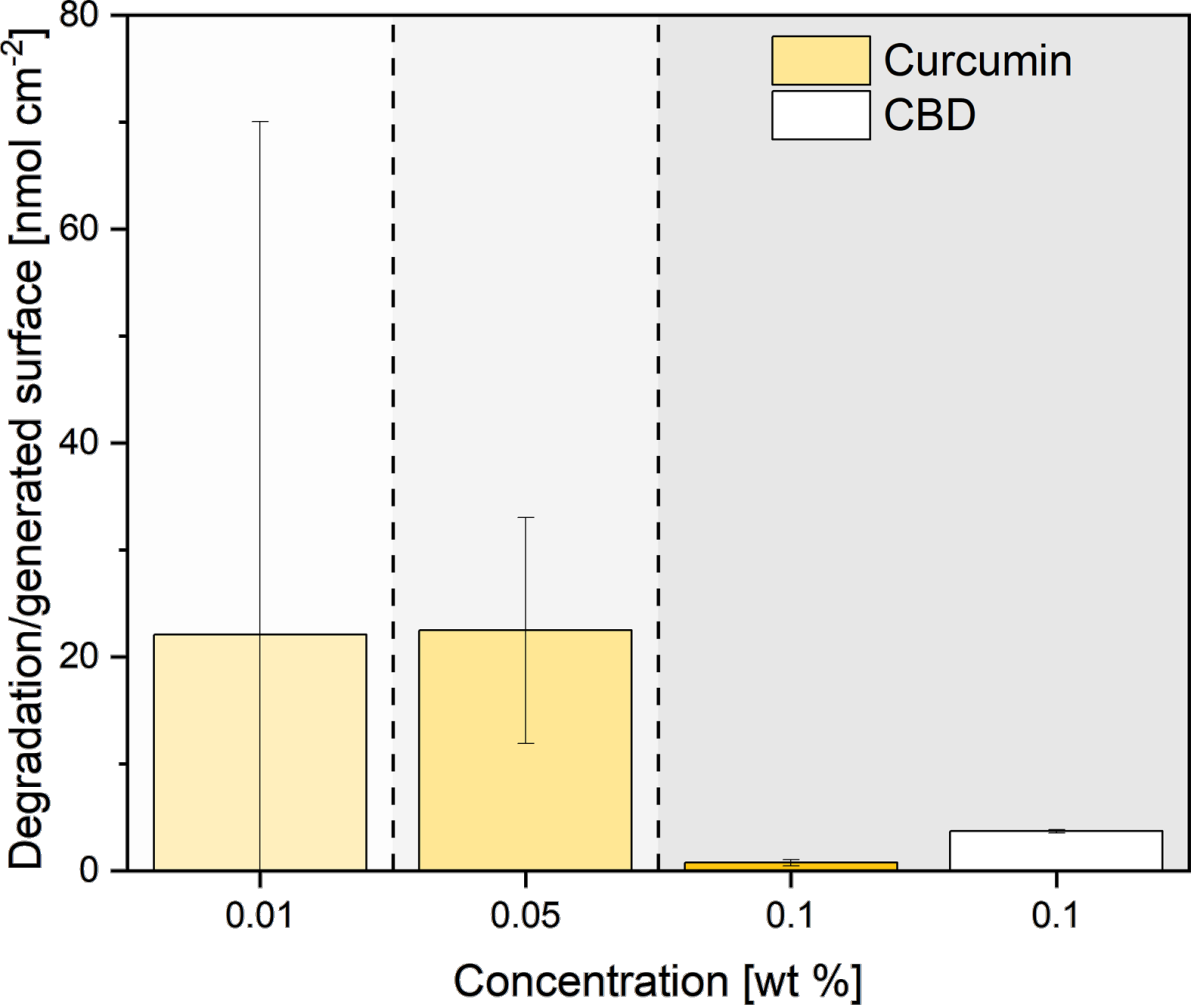
Amount of degradation products normalized to the particle surface generated via MP-LFL after one laser passage for different concentrations of curcumin (yellow bars) and CBD (white bar). The bar charts depict the mean values of three independent measurements (*n* = 3) with the standard deviation shown as error bars.

Based on the previous findings, it may be concluded that an increase in fragmentation efficiency and a higher yield of SMPs always coincides with a higher degree of chemical degradation. Hence, further process optimization is necessary to quantitatively compare the interplay between these opposing mechanisms (fragmentation versus chemical degradation). The main advantage of fragmentation is a reduction of particle size, which goes along with an elevated specific surface area that improves solubility and bioavailability of these nutraceuticals. Consequently, the amount of degradation products per generated surface area within one laser passage is a particularly interesting assessment criterion for process efficiency. Moreover, the created surface area is also a descriptor highly relevant for application (as the solubility of hydrophobic particle dispersions in water is proportional to the surface [67–68]). For simplified calculations of the total surface created by LFL, we assumed all particles to be ideal spheres. Now, the generated by-products may be normalized to the product particle surface (in other words, unintended per intended effects), which is depicted in Figure 6. In the 0.01 wt % curcumin sample, the amount of impurities per generated surface area was 22 nmol·cm^−2^. When the concentration was increased tenfold to 0.1 wt %, the value decreased to 0.8 nmol·cm^−2^, mainly driven by the overall tenfold reduction of degradation when switching to a higher particle mass concentration. Based on these findings it may be concluded that for surface area generation at minimized degradation, a higher curcumin mass concentration is beneficial.

Regarding material-specific effects, when comparing the rate of degradation per specific surface area caused for curcumin and CBD at the same mass concentration of 0.1 wt %, it is noticeable that CBD with 3.7 nmol·cm^−2^ yields significantly more surface-specific degradation products than curcumin with 0.8 nmol·cm^−2^ (Figure 6). This is presumably ruled by the lower melting/degradation temperature [50,69], or by the larger particle size of CBD. The determined values for the corresponding samples can be found in Tables S5 and S6, Supporting Information File 1. Nevertheless, not only the direct comparison of the produced SMP fractions of curcumin and CBD (Figure 3 and Figure 4), as well as the different yields (Figure 2), but also differences in the formation of degradation products per gained specific surface show very clearly that there must be a material- or size-dependent difference. In the material systems examined here, this difference can be attributed either to the absorption coefficient of the bulk material or the diameter of the educt particles, a correlation more closely examined in the consecutive section.

### Proposed MP-LFL mechanism

While both commercially available nutraceuticals (curcumin and CBD) are polycrystalline, nearly water-insoluble natural products with a similar molecular structure (Figure 1) and approximately the same molecular weight of 314 g·mol^−1^ for CBD and 368 g·mol^−1^ for curcumin, already their visual appearance strongly deviates. CBD is colorless with an extinction band far in the UV at 210 nm [70]. Curcumin, in contrast, is known for its typical deep yellow color derived from a wavelength extinction maximum at 425 nm [57], which is close to the wavelength (532 nm) of the used laser. Hence, differences in both the material-specific molar extinction and particle-related extinction cross sections are conceivable. To differentiate molecular extinction properties from those of the particles, both suspensions were transformed to their molecular state by dissolution. Figure S13, Supporting Information File 1, shows the extinction spectra of curcumin and CBD samples dissolved in acetonitrile at the same mass concentration in the spectral range from 190 to 900 nm. Both substances show no relevant extinction at the laser wavelength of 532 nm (2.33 eV), but at the two-photon energy corresponding to a wavelength of 266 nm (4.66 eV) the curcumin molecule has higher absorption values than CBD. Multiphoton absorption has been discussed to be responsible for the 523 nm picosecond-LFL of polymer-capped gold nanoparticles [71]. Based on these findings, laser absorption cannot be the only reason for the elevated fragmentation efficiency in curcumin, unless multiphoton absorption is responsible for the higher LFL efficiency. A further obvious difference between the two model substances is the educt particle size, which strongly affects the extinction of the laser light, while particularly in MP-containing samples the ratio of absorption and Mie scattering needs to be carefully considered. The SEM images in Figure 3A and the number-weighted particle size distribution of the curcumin feedstock material in Figure 3C show a bimodal size distribution mainly composed of two size fractions, a smaller main fraction with a modal value of *x*_mode 1, LP0_ = (1.04 ± 0.05) µm, and a larger size fraction with a modal value of *x*_mode 2, LP0_ = (5.06 ± 0.29) µm. CBD, as shown in Figure 4A,C, also has a small proportion of smaller particles with a modal value of *x*_mode 1, LP0_ = (3.56 ± 0.43) µm, a recognizable main mode of *x*_mode 2, LP0_ = (9.08 ± 0.37) µm, as well as some significantly larger particles (>10 µm), which cannot be quantified in the analytical centrifuge (which is why we additionally analyzed CBD using SEM images, Figure S6, Supporting Information File 1). Modal values for all concentrations and substances are listed in Tables S3 and S4, Supporting Information File 1.

**Figure 7:**
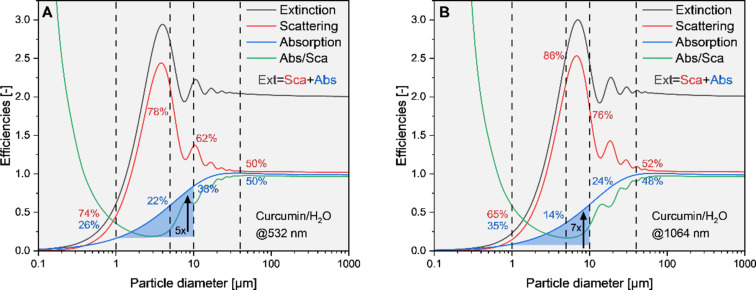
Size-dependent ratio and efficiency of extinction for curcumin, based on Mie-plot calculations for wavelengths of 532 nm (A) and 1064 nm (B), based on a refractive index and an absorption coefficient of curcumin of, respectively, 1.42 and 0.01 taken from literature [73–74]. Dashed lines indicate the particle diameters of interest (1, 5, 10, and 40 µm), and the numbers correspond to the relative contributions of absorption and scattering to extinction.

For curcumin, the influence of particle size on extinction, absorption, and scattering was calculated based on the Mie theory (using the program MiePlot [72]) for spherical particles in a range of 0.1 to 1000 µm at wavelengths of 532 and 1064 nm (Figure 7). For monodisperse particle sizes (1, 5, 10, and 40 µm) the relative ratio of absorption to scattering was calculated from Figure 7, and a clear size dependency was observed. Considering the relative values, it is noticeable that the portion of the fragmentation-relevant absorption efficiency (Figure 7, blue graph and values) of the extinction (Figure 7, black graph) differs with increasing particle size from 1 to 40 µm. For a wavelength of 532 nm, starting from 26% for 1 µm particles, the portion of absorption initially drops to 22% when reaching a particle diameter of 5 µm. For 10 µm spheres, in contrast, the absorption efficiency percentage increases again to 38% and subsequently reaches 50% for 40 µm particles, determined from the curves depicted in Figure 7. However, Figure 7 also shows that the fragmentation-relevant absolute absorption increases continuously with increasing particle size. For 532 nm, a tenfold increase in particle size (from 1 to 10 µm) leads to an absolute absorption enhancement by a factor of 5, for 1064 nm by a factor of 7 (Figure 7, shown in blue). The absorption/scattering ratio shown in green in Figure 7 also shows that the absorption fraction is significantly higher for SMPs and decreases with increasing SMP size. For MP-LFL of curcumin, as illustrated in the particle size distribution in Figure 3, the particle size range above the SMPs is particularly relevant, showing that for small MPs between 1 and 5 µm, the absorption/scattering ratio decreases and increases again from 5 µm, which preferentially leads to comminution of particles larger than 5 µm. To substantiate the theoretical data with experimental approaches, experimentally generated curcumin particle ensembles were measured in a double integrating sphere at a laser wavelength of 1064 nm. The experimental data provides a ratio of absorption to scattering of 31%:69%, which is in good agreement with the theoretical observations in Figure 7. This leads to the hypothesis that the fragmentation of MPs is preferred in larger particles ≫1 µm, as they absorb laser energy more efficiently, which is consistent with the observations of the particle size shift in curcumin and CBD (Figure S6, Supporting Information File 1). Based on these findings it may be concluded that the formation of organic SMP and NP fractions by single-pulse LFL in the examined material systems curcumin and CBD is primarily ruled by two effects, namely, (I) an increase in laser energy absorption with particle size, which makes larger particles more susceptible to fragmentation, and (II) an increased LFL efficiency by multiphoton absorption.

These considerations coincide with our mechanistic fragmentation hypothesis already discussed for organic drugs [43] and inorganic IrO_2_ [14] based on shock wave formation caused by stress confinement. The conditions for stress confinement are fulfilled if the pulse duration is shorter than the acoustic relaxation time and thus a maximum pressure increase occurs in the particle. For curcumin and CBD, this is the case with a pulse duration of 10 ps (as the acoustic relaxation time is higher, *t*_ac, 1 µm_ ≥ 500 ps, Equations S11 and S12, Supporting Information File 1). Depending on the optical penetration depth, the particle size influences whether homogeneous or inhomogeneous heating takes place. The heated volume leads to the formation of thermoelastic stress and finally to the formation of shock waves, promoting mechanical fragmentation. For curcumin, the mean optical penetration depth is 32 µm (*d*_opt, 1 µm_ = 39 µm, *d*_opt, 10 µm_ = 27 µm), and for CBD, the mean optical penetration depth is 170 µm. The mean absorption coefficients of 309 and 59 m^−1^, respectively (assuming the same ratio of absorption of curcumin and CBD, Figure S14, Supporting Information File 1), were experimentally determined by Beer–Lambert calculations of the attenuation of the laser light and the measured ratio of absorption in the double integrating sphere. For both material systems, the mean optical penetration depth is well above the average educt particle size and therefore leads to homogeneous heating of the educt by the laser. Overall, the nutraceutical particles are homogeneously heated during picosecond-LFL, and the criterion for photomechanical effects is fulfilled, causing shock wave formation. Analogously to our previous works on organic drugs [43] and on inorganic oxide MPs [14], it is again shown that the dominant fragmentation effect is due to photomechanical comminution and that thermal effects are negligible.

### Biocompatibility and antioxidant properties of laser-fragmented curcumin

**Figure 8:**
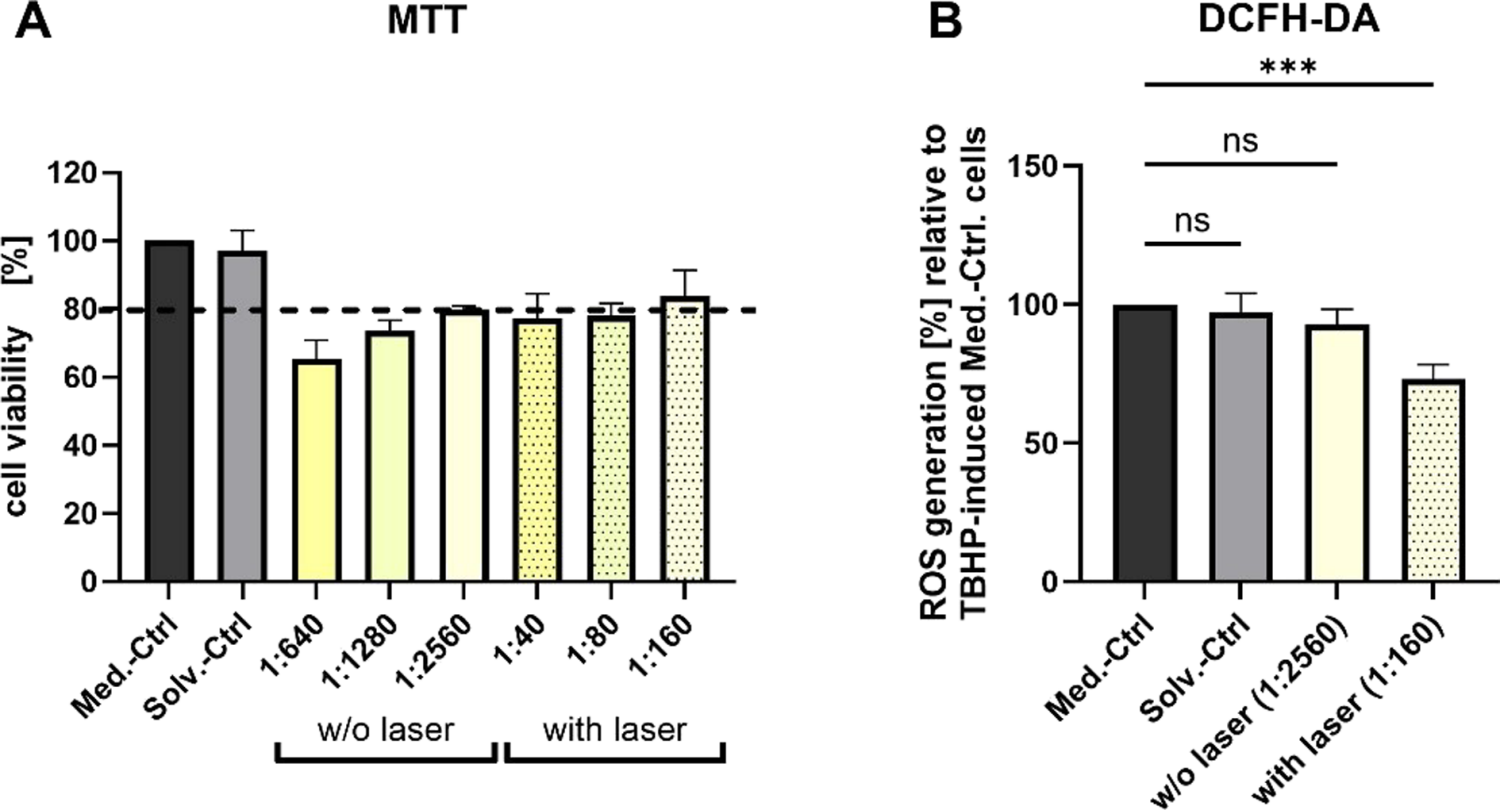
Biocompatibility and antioxidant capacity of laser-fragmented (“with laser”, LP1) and unirradiated control (“w/o laser”, LP0) curcumin samples in HepG2 cells using MTT (A) and DCFH-DA (B) assays, respectively. (A) Different dilutions of 0.5 g·L^−1^ LP1- and LP0-irradiated curcumin stock solutions were tested in HepG2 cells. Cell viability was assessed with the MTT assay after 24 h treatment time. 80% cell viability was set as the cut-off for a non-cytotoxic effect (dashed line) in relation to the medium control (Med.-Ctrl). 20% water-diluted medium served as solvent control (Solv.-Ctrl). Shown are mean values with the standard deviation as error bars, *n* = 3, biological replicates. (B) Non-cytotoxic dilutions of LP1- and unirradiated curcumin samples were tested in the DCFH-DA assay for their capacity to reduce reactive oxygen species (ROS) in oxidatively stressed HepG2 cells in relation to the medium control. 0.025% water-diluted medium served as solvent control. Shown are mean values with the standard deviation as error bars, *n* = 3, biological replicates. Asterisks indicate the significance of differences between differently treated cells determined by one-way analysis of variance (1-way ANOVA) followed by Dunnett’s multiple comparisons test with a significance level α of 0.05. Three asterisks indicate values of *P* ≤ 0.001. Values of *P* > 0.05 were marked as not significant (“ns” in the graph).

As LFL of curcumin was more efficient than that of CBD, curcumin was tested for its cytotoxic and antioxidant potential in an in vitro cell culture model. The hepatocellular carcinoma cell line HepG2 was chosen for analyzing the antioxidant capacity of LP1-irradiated curcumin compared to the unirradiated LP0 control. Non-cytotoxic concentrations for both curcumin samples were determined using the MTT assay first. The cell viability was compromised for the untreated curcumin even at high sample dilution, whereas the laser-fragmented curcumin showed high biocompatibility. In detail, a threshold viability of 80% was reached at a 1:2560 dilution (0.195 mg·L^−1^) for the unirradiated sample; the tolerance for the laser-fragmented curcumin samples, however, was ca. 30 times higher even at a lower dilution of 1:160 (6.25 mg·L^−1^) (Figure 8A). The main difference between the samples are the higher specific surface areas in the irradiated curcumin dispersions, as well as a potential decrease in particle crystallinity. Both effects are known to increase the water solubility of curcumin, yielding higher total concentrations of dissolved active and bioavailable curcumin molecules [75–76]. The effect of curcumin on cancer cells has been frequently examined, and two potentially contradictory effects are frequently discussed. On the one hand, curcumin nanoparticles are said to be more cytotoxic to cancer cells like HepG2 than the corresponding microparticle formulations [77]. On the other hand, antioxidant features of curcumin are also frequently discussed [78–79], which may overshadow toxic effects and improve cell viability at higher concentrations of active soluble curcumin. The second protective effect seems to be dominant in our study, which may be attributed to the fact that the laser-fabricated curcumin formulations do not require any artificial stabilizers or solvents, minimizing unwanted toxicological side effects that cannot be ruled out in most other studies. To further test the capacity of laser-fabricated curcumin formulations in reducing the amount of ROS in artificially oxidatively stressed cells, we conducted a dichlorodihydrofluorescein diacetate (DCFH-DA) assay at the corresponding maximum non-cytotoxic concentration based on Figure 8A. Higher concentrations of LP1-irradiated curcumin could be applied in the DCFH-DA assay. We can see a substantial reduction of ROS generation in oxidatively stressed cells by 27% ± 5% in laser-irradiated cells in comparison to the non-irradiated controls (Figure 8B), which hints towards the prevalence of antioxidant effects in this work and underlines the prospects of curcumin LFL for anti-ROS applications.

## Conclusion

LFL is an emerging field in the contamination- and degradation-free comminution of organic MPs to SMPs and NPs, aiming at an increase in the specific surface area and a corresponding solubility enhancement to improve the oral resorption of poorly bioavailable drugs such as naproxen, prednisolone, ketoconazole, megestrol acetate [43], or vitamin C [35], as well as nutraceuticals such as capsaicin [35], curcumin [80], and CBD. Here, modern flow-through reactors are particularly interesting as they allow for a systematic variation of scaling key factors and help to elevate comminution efficiency at low chemical degradation. For a reasonable scale-up of this process in a continuous operating mode, suitable irradiation conditions within a single process step are required. Single-PPV conditions allows one to consider simple process intermediates and derive scaling conclusions; linear throughput increase can then be achieved by matching the flow rate with the laser repetition rate. In this work, we investigated the requirements for the efficient generation of organic SMPs with diameters <1000 nm from MP suspensions under near single-PPV conditions, varying mass concentration and particle size of the educt dispersions. To this purpose, the nutraceutical model substances curcumin and CBD were irradiated with a green picosecond laser in a CJ flow-through reactor. Our results show that even a single pulse leads to fragmentation of both MP materials, whereby less concentrated suspensions tend to form relatively more SMPs (i.e., higher yield). However, the interplay between a higher particle mass throughput through elevated concentrations and the loss in particle yields due to Beer–Lambert attenuation effects requires more thorough future investigations. Furthermore, the choice of the ideal educt particle diameters was investigated under one-PPV conditions. Here, the key is to use feedstock particle diameters of 5–10 µm as there is an interplay between two opposing effects, namely, (I) an increased absorbance-to-scattering ratio of laser light by larger MPs, which benefits efficient particle fragmentation, and (II) differences in the MP dissociation pathways, which may hinder the formation of the desired SMPs and NPs when educt particles are too large. For both MP material systems we confirmed, based on the calculation of the acoustic relaxation time, that the stress confinement condition for the use of picosecond lasers is fulfilled; thus a contribution of photomechanical comminution is highly probable and thermal effects are negligible. Determinants for the by-product formation were discovered with an elevated absolute degradation at lower concentrations, but a lower degradation per created surface area. Hence, selectivity for the desired SMPs over the by-products was maximal for curcumin at high concentrations of 0.1 wt %, with 0.8 nmol·cm^−2^ degradation product per surface area produced (compared to 3.7 nmol·cm^−2^ for CBD at the same concentration). Note that, in all cases, the amount of degradation products was significantly lower compared to established methods like cryo-milling and comparable to the quantities found in LFL of drugs [43]. To realize an industrially relevant scale-up, recent curcumin LFL experiments [80] carried out in a specialized flat jet flow-through reactor showed better control of the laser fluence and more precise PPV adjustment, allowing for the use of significantly higher concentrations (because of the low liquid layer thickness) [81]. Also, to gain deeper insights into the fragmentation mechanisms of organic MPs, pump–probe microscopy imaging, as already performed for the LFL of inorganic microparticles, which showed photomechanical contributions to the LFL mechanism as well [14], is mandatory to verify the previously discussed contribution of photoacoustic comminution based on tensile wave formation. Furthermore, our findings highlight the presence of antioxidant effects when laser-fragmented curcumin particles are exposed to cancer cell lines, which is well in line with other curcumin formulations but highlights that LFL is a promising comminution strategy to generate bioactive curcumin in the absence of additional stabilizing ligands and additives.

## Experimental

### Materials

Curcumin from turmeric rhizome (95%) was purchased from Thermo Fisher GmbH. Cannabidiol (≈99%) was provided by “Ott im Pott – CBD Shop Essen”. Acetonitrile, potassium dihydrogen phosphate, and orthophosphoric acid (>85%) for HPLC analysis were purchased in HPLC grade from VWR International GmbH. All aqueous solutions were prepared with deionized water obtained by using a Milli-Q system from Merck Millipore.

### Methods

#### Preparation of MP suspensions

Different concentrations of curcumin (0.01, 0.05, and 0.1 wt %) and CBD (0.1, 0.5, and 1 wt %) were prepared as aqueous suspensions in deionized water and dispersed three times for 5 s with an ultrasonic sonotrode (Hielscher Ultrasonics GmbH).

#### Pulsed laser fragmentation in liquids

For MP-LFL, an Edgewave PX400-3-GH laser (EdgeWave GmbH) with a wavelength of 532 nm, a pulse duration of 10 ps, and an average power of 10 W was used. In the established CJ setup (Figure 1) [12–13,15,82], a repetition rate of 100 kHz ensured that each volume element was hit by 1.6 ± 0.4 pulses (exact calculation in Table S1, Supporting Information File 1). However, the real PPV number (= equal to pulses per particle) in the CJ may differ by a factor of up to two because of the laminar flow conditions between the edge and the center at a linear flow velocity of 1.53 m·s^−1^ and the Reynolds number of 1830, which is well within the laminar regime of a free liquid-jet reactor [83]. Hence, a certain PPV variance around the nominal value of 1 PPV cannot be excluded, but the majority of the volume is likely treated at approx. 1 PPV. For a quantitative statement on the variance, residence time measurements would be required. In the setup, a plano-convex cylindrical lens with a focal length of 100 mm was used to focus the laser on the liquid jet, resulting in an average incidence fluence of (284 ± 45) mJ·cm^−2^. The estimated fluence values are defined as the incident fluence at the CJ interface. Of course, in the excited volume there are geometry-related fluence gradients due to refraction at the curved interface between ambient air and the liquid jet, as well as concentration effects attenuating the laser beam, which will be considered in the discussion. Details on the calculations of fluence, PPV, and Reynolds numbers can be found in Supporting Information File 1 (Equations S1–S7), while values used in these calculations are listed in Tables S1 and S2, Supporting Information File 1.

#### UV–vis extinction spectroscopy

A UV–vis/NIR spectrometer UV3600i Plus (Shimadzu Deutschland GmbH) was used to determine the increase in extinction of curcumin and CBD. To this purpose, the fragmented samples LP1 as well as the references LP0 of different concentrations were decanted for 12 h to remove large particles, and then the supernatant was analyzed. To dissolve the particles, 1 mL of the supernatant was mixed with 1 mL of acetonitrile and then measured using a UV–vis extinction spectrometer. The extinction maximum of 417 nm was evaluated for curcumin, and the extinction maximum for CBD was 207 nm. The ratio of LP1 to LP0 results in the percentage extinction enhancement that is shown in Figure 2A for curcumin and Figure 2B for CBD.

#### High-performance liquid chromatography

The purity of curcumin and CBD was analyzed using a Nexera SCL-40 liquid chromatography system with a DGU-403 degasser unit, SIL-40C XR autosampler, and SPD-M30A UV detector from Shimadzu Deutschland GmbH. A 100 mm Nucleosil-C18 column incl. precolumn from Macherey-Nagel GmbH & Co. KG with an inner diameter of 4 mm and particle sizes of 3 µm was used. For curcumin, the mobile phase consisted of a 1.36 g·L^−1^ solution of potassium dihydrogen phosphate previously adjusted to pH 2.0 with phosphoric acid (A) and acetonitrile (B). The initial concentration of mobile phase B is increased from 30% to 70% within the time interval from 0 to 8 min, then further increased to 90%; this concentration is maintained for 2 min and then reduced to the initial concentration of 30% and maintained for 2 min. All samples were mixed with acetonitrile at a ratio of 3:5 to dissolve the curcumin particles. Aliquots of 10 µL were injected at a flow rate of 1.0 mL·min^−1^ at 40 °C. The chromatograms were recorded over 12 min at a wavelength of 425 nm (Figures S7–S9, Supporting Information File 1). The mobile phase for CBD consists of 25% deionized water and 75% acetonitrile. To dissolve the CBD samples, all concentrations were mixed 1:1 with acetonitrile. Aliquots of 1 µL (for 1 wt %) or 10 µL (for 0.1 and 0.5 wt %) of the samples were injected at a flow rate of 1.5 mL·min^−1^ at 30 °C. The chromatograms were recorded at 214 nm for 6 min (Figures S10–S12, Supporting Information File 1). All samples were filtered through 0.2 µm syringe filters before insertion into the autosampler. To identify degradation products, the LP0 references of curcumin and CBD were measured in addition to the fragmented LP1 samples. All additional peaks that occurred in the fragmented samples and not in the untreated control were assumed to be degradation products. The amount of the pure substance and the degradation products of the laser-generated samples were determined in percentage terms by calculating the area under the curve (AUC).

#### Analytical centrifuge

The particle size analysis of CBD was determined using a LUMiSizer 651 dispersion analyzer (LUM GmbH). The number-weighted particle size distribution was determined using sedimentation profiles according to ISO 13318-3 [84]. Before the measurement, untreated and laser-generated CBD suspensions of all concentrations were redispersed in an ultrasonic bath. The sedimentation profiles were measured at a wavelength of 410 nm and a temperature of 25 °C using a velocity ramp that was increased from 300*g* to 4000*g*. For the evaluation, a method was developed that enables different substances and concentrations to be compared in terms of comminution success. A similar method has already been described by Friedenauer et al. for volume-weighted determinations [43]. By integrating the number-weighted particle density distribution (*q*_0_(*x*)), the AUC for particles <1000 nm could be determined and normalized to the total AUC of all particles with a cut-off diameter of 10000 nm (LP*x* fraction < 1000 nm = AUC_<1000 nm_/AUC_total_) to ensure comparability between samples. From the difference between LP1 and LP0, the SMP fraction produced can be calculated, which allows for a quantitative statement about the normalized proportion of particles in the submicrometer range compared to the untreated LP0 sample. It should be noted here that the LUMiSizer can quantitatively characterize particle suspensions in the range of 100–10000 nm, depending on the used sedimentation protocols; size fractions outside this range cannot be reliably quantified.

#### Scanning electron microscopy

SEM images were produced to determine the particle size and shape of curcumin and for CBD. Different magnifications of a sample were recorded, and at least 500 particles were evaluated to depict the polydisperse sample sufficiently with the image processing program ImageJ. The samples were added onto Si wafers, evaporated in a drying oven, and sputtered (80% Au + 20% Pd). An Apreo S LoVac-SEM (Thermo Fisher GmbH) was used. The calculation of LP*x* fraction < 1000 nm from the quotient of AUC_<1000 nm_/AUC_total_, as well as the previously described SMP fraction from the difference of LP1 fraction < 1000 nm to LP0 fraction < 1000 nm for curcumin was carried out analogously to the CBD calculations described in the previous section.

#### Double integrating sphere

The setup consisted of two golden integrating spheres, a measuring chamber (FTIR-600, Linkam Scientific Instruments LTD), a 1064 nm laser (Roithner Lasertechnik GmbH), and two photodiodes as power sensors (PowerMax-USB UV/VIS Quantum Sensors, Coherent Inc.). The measuring chamber had two protective glasses of BaF_2_ (thickness of 0.5 mm, refractive index of 1.45, transmittance of more than 90% at 1064 nm) at the top and the bottom. The powder samples were pressed onto a thinner BK7 glass (thickness of 0.17 mm, refractive index of 1.5, transmittance of more than 90% at 1064 nm), which was placed inside the processing chamber. The laser beam had a Gaussian profile with a beam diameter of (1.2 × 1.3) mm^2^. A laser output power of about 80 mW was chosen for the optical experiments. The laser beam interacted with the powder at five different positions on the sample for 3 s. Measurements were taken five times on fresh material. From the measured transmitted and reflected power, the transmittance (Trans) and reflectance (Sca) of the laser radiation were determined. The exact formulas and their derivations can be found elsewhere [85]. The absorptance (Abs) was then calculated as the difference between 100% and the transmittance and reflectance/scattering (Abs = 100% − Sca − Trans).

#### MTT assay

About 5·10^4^ HepG2 cells were seeded per well in triplicates for each test solution in a 96-well plate in 200 µL Dulbecco’s Modified Eagle’s medium (DMEM) supplemented with 10% (v/v) fetal bovine serum and 1% (v/v) penicillin/streptomycin (standard medium) in a humidified atmosphere containing 5% CO_2_ at 37 °C. After 24 h of incubation, the medium was removed, and the cells were treated with 100 µL standard medium containing different amounts of LP1- and LP0-irradiated curcumin. Therefore, the 0.5 g·L^−1^ stock dispersion was serially diluted (twofold) with standard medium starting with a 1:5 dilution and ending with dilutions of 1:2560 (LP0 samples) and 1:160 (LP1 samples). Cells were treated for 21 h followed by MTT ((3-(4,5-dimethylthiazol-2-yl)-2,5-diphenyl-2*H*-tetrazolium bromide) addition (5 µL of MTT stock solution, *c* = 5 mg·mL^−1^ in phosphate-buffered saline). After 3 h, cells were lysed and Formazan, formed by living cells, was dissolved via addition of 100 µL solubilization solution (10% sodium dodecyl sulfate in 0.01 M HCL) over night. Formazan was colorimetrically quantified at 570 nm and mean values for technical triplicates were calculated. Cell viability was assessed by setting the mean absorption values for each test solution in relation to the non-treated control value (medium control). The medium control was set to 100%.

#### DCFH-DA assay

About 2·10^4^ HepG2 cells were seeded per well in triplicates for each test solution in a 96-well plate in 200 µL DMEM standard medium in a humidified atmosphere containing 5% CO_2_ at 37 °C. The next day, medium was removed, and cells were treated with LP1- and LP0-irradiated curcumin at the lowest non-cytotoxic dilutions (LP1 = 1:160 and LP0 = 1:2560 according to the MTT assay) in 200 µL standard medium for 24 h. Subsequently, cells were stressed with 0.5 mmol·L^−1^ *tert*-butyl hydroperoxide for 2 h followed by 2′,7′-dichlorodihydrofluorescein diacetate (DCFH-DA, *c* = 5 µmol·L^−1^) addition for 30 min. The non-fluorescent and cell-penetrating DCFH-DA is oxidized via ROS to fluorescent 2′,7′-dichlorofluorescein, which can be colorimetrically quantified at 525 nm after excitation at 485 nm. Thus, mean values of technical triplicates for each test solution indirectly indicate cellular ROS levels. Relative ROS reduction was calculated by dividing the fluorescent intensities of the test wells by one of the stressed control cells (medium control) without further treatment. The medium control was set to 100%.

#### Statistical analysis

The bar charts display the average values of at least three independent measurements (*n* ≥ 3). The standard deviation is indicated by the error bars. Stars mark the significance of differences between the three different concentrations (0.01, 0.05, and 0.1 wt % or 0.1, 0.5, and 1.0 wt %), which were determined by one-factor analysis of variance (1-way ANOVA) with a significance level α of 0.05. Values of *P* ≤ 0.05 are summarized with one star, values of *P* ≤ 0.01 are summarized with two stars, and values of *P* ≤ 0.001 are given with three stars. Values of *P* > 0.05 were marked as not significant (“ns” in the graphs). For cell culture a Dunnett’s multiple comparisons test followed 1-way ANOVA.

## Supporting Information

Supporting Information contains calculations of laser parameters and flow profiles (Reynolds number), additional information on LFL-induced extinction increase of curcumin and CBD, information on particle size distribution of curcumin and CBD before and after LFL, HPLC spectra, information on selectivity, information on optical properties of curcumin and CBD, calculations on stress confinement and resulting temperature and phase diagrams, as well as calculations on SMP yield and productivity.

File 1 Additional experimental data.

## Data Availability

All data that supports the findings of this study is available in the published article and/or the supporting information of this article.
