# Supplementary material for: Efficiency of single-pulse laser fragmentation of organic nutraceutical dispersions in a circular jet flow-through reactor
Source: Beilstein J Nanotechnol. 2025 May 26;16:711–27. doi: 10.3762/bjnano.16.55 (PMC12130626; doi:10.3762/bjnano.16.55)
Supplement: File 1 — Additional experimental data. [file Beilstein_J_Nanotechnol-16-711-s001.pdf]

BEILSTEIN JOURNAL OF NANOTECHNOLOGY

Supporting Information

for

Efficiency of single-pulse laser fragmentation of organic nutraceutical dispersions in a circular jet flow-through reactor

Tina Friedenauer, Maximilian Spellauge, Alexander Sommereyns, Verena Labenski, Tuba Esatbeyoglu, Christoph Rehbock, Heinz P. Huber and Stephan Barcikowski

Beilstein J. Nanotechnol. **2025,** *16,* 711–727. doi:10.3762/bjnano.16.55

Additional experimental data

License and Terms: This is a supporting information file under the terms of the Creative Commons Attribution License (https://creativecommons.org/licenses/by/4.0). Please note that the reuse, redistribution and reproduction in particular requires that the author(s) and source are credited and that individual graphics may be subject to special legal provisions.
The license is subject to the *Beilstein Journal of Nanotechnology* terms and conditions: (https://www.beilstein-journals.org/bjnano/terms)

Calculation of the mean fluence and PPV.

The vertical radius of the raw laser beam, $\omega_{0,\mathrm{vert}}$, the mean power, $\bar{P}$, the volumetric flow rate, $\dot{V}_{\mathrm{fluid}}$, and the area of the nozzle within the jet, A_{nozzle}, were determined experimentally. All values shown in Table S1 were calculated using the following Equations (S1–S6):

$$w_f = \frac{h_{spot}}{2} = \frac{\lambda * f * M^2}{\pi * \omega_0} \quad \text{(S1)}$$

$$A_{\mathrm{spot}} = \pi * \omega_{0,horiz} * \omega_{\mathrm{f}} \quad \text{(S2)}$$

$$E_{pulse} = \frac{P_{mean}}{R} \quad \text{(S3)}$$

$$F_0 = \frac{E_{pulse}}{A_{\mathrm{spot}}} \quad \text{(S4)}$$

$$v_{\mathrm{fluid}} = \frac{\dot{V}_{\mathrm{fluid}}}{A_{\mathrm{nozzle}}} \quad \text{(S5)}$$

$$\mathrm{PPV} = R * \left(\frac{h_{\mathrm{spot}}}{v_{\mathrm{fluid}}}\right) \quad \text{(S6)}$$

Table S1: Laser and process parameters used to calculate the fluence and the pulses-per-volume element (PPV).

**Parameter**		**Value**	**Unit**	**Ref.**
λ	Wavelength	532	nm	-
f	Focal length	100	mm	-
M^2	Beam quality	1.3	-	-
t_p	Pulse duration	10	ps	-
$\omega_{0,\text{ horiz}}$	Horizontal raw beam radius	904 ± 50	µm	Exp.
$\omega_{0,\text{ vert}}$	Vertical raw beam radius	1815 ± 50	µm	Exp.
$\omega_{f,\text{ measured}}$	Focused beam radius, meas.	13 ± 1	µm	Exp.
$\omega_{f,\text{ calculated}}$	Focused beam radius, calc.	12 ± 1	µm	Eq. S1
$h_{\text{spot, measured}}$	Laser spot height, meas.	26 ± 1	µm	Exp.
$h_{\text{spot, calculated}}$	Laser spot height, calc.	24 ± 1	µm	Eq. S1
P_{mean}	Mean power	9.8 ± 0.4	W	Exp.
R	Repetition rate	100	kHz	-
E_{pulse}	Pulse energy	98 ± 10	µJ	Eq. S3
A_{spot}	Spot size	34447 ± 4220	μm^2	Eq. S2
F_0	Mean incident fluence	284 ± 45	mJ cm^{-2}	Eq. S4
$\dot{V}_{\text{fluid}}$	Volumetric flow rate	1.73 ± 0.03	ml s^{-1}	Exp.
A_{nozzle}	Area nozzle (circular area with *d* = 1.2 mm)	$1.13 \cdot 10^{-6}$ ± $1.88 \cdot 10^{-7}$	m^2	-
v_{fluid}	Linear fluid velocity	1.53 ± 0.26	m s^{-1}	Eq. S5
**PPV**	Pulses-per-volume element	1.6 ± 0.4	-	Eq. S6

Calculation of the Reynolds number to determine the flow profile.

$$\mathrm{Re} = \frac{\rho_0 v_{fluid} d}{\eta_{fluid}} \qquad \text{(S7)}$$

Table S2: Parameters and values used to calculate the Reynolds number for CJ.

Parameter		Value	Unit	Ref.
ρ_0	Fluid density (25 °C)	997	kg m^{-3}	[1]
v_{fluid}	Linear fluid velocity	1.53±0.26	m s^{-1}	Eq. S5
d	Charac. length (inner diameter)[2–4]	1.2*10^{-3}	m	Exp.
η_{fluid}	Dynamic fluid viscosity (20 °C)	1*10^{-3}	Pa s	[1]
Re_{CJ}	Reynolds value CJ	1830	-	Eq. S7

Extinction enhancement after laser treatment.

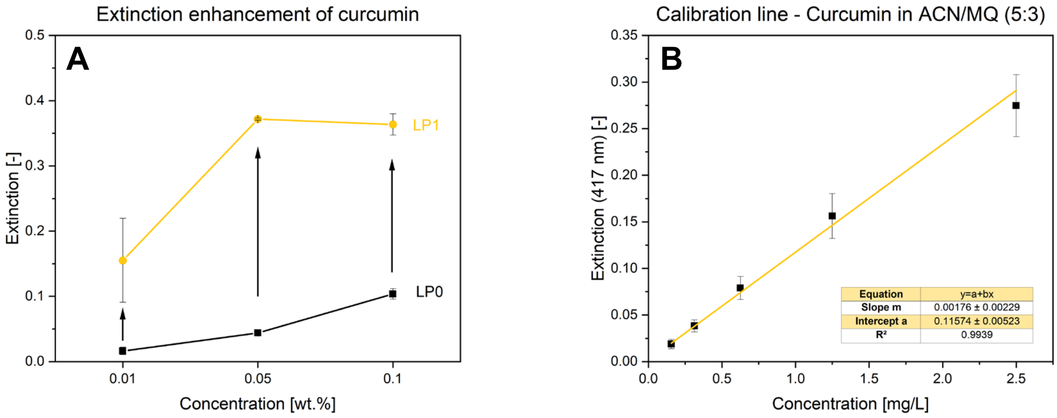

Figure S1: Extinction enhancement (A) and calibration line to determine the SMP concentration (B) of curcumin.

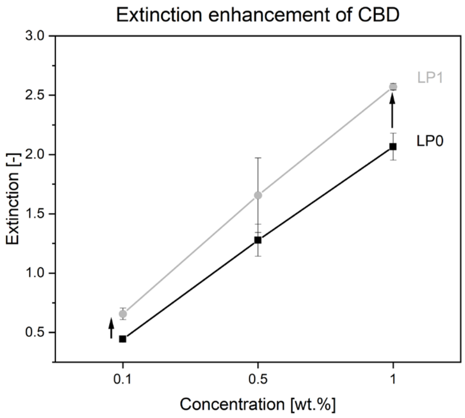

Figure S2: Extinction enhancement of CBD.

Number-weighted particle size distribution of curcumin and CBD.

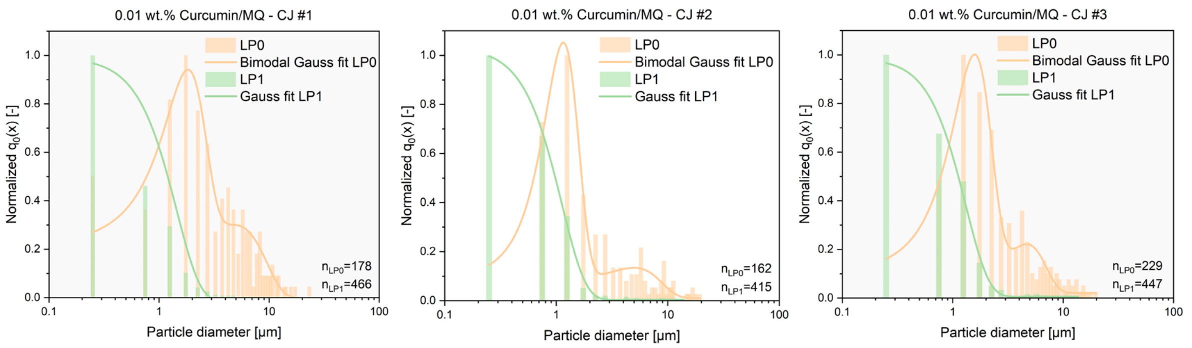

Figure S3: Particle size reduction of 0.01 wt % curcumin in the CJ at 532 nm after LP1 (LFL), three independent determinations via SEM and ImageJ.

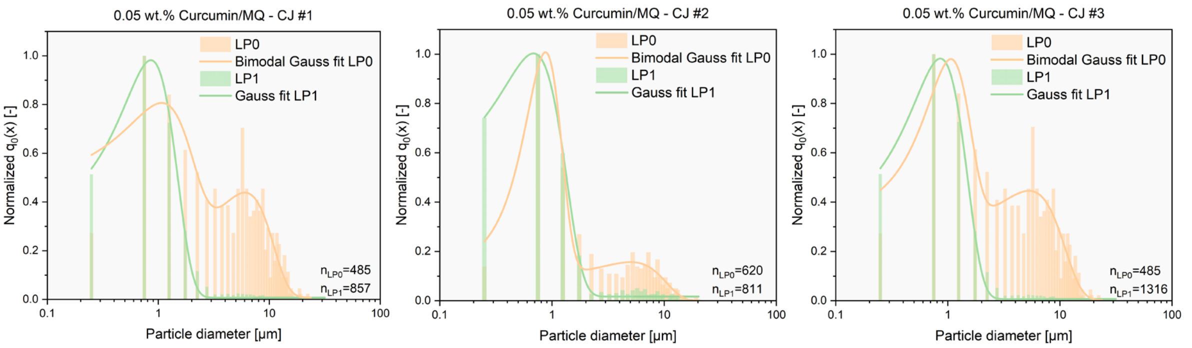

Figure S4: Particle size reduction of 0.05 wt % curcumin in the CJ at 532 nm after LP1 (LFL), three independent determinations via SEM and ImageJ.

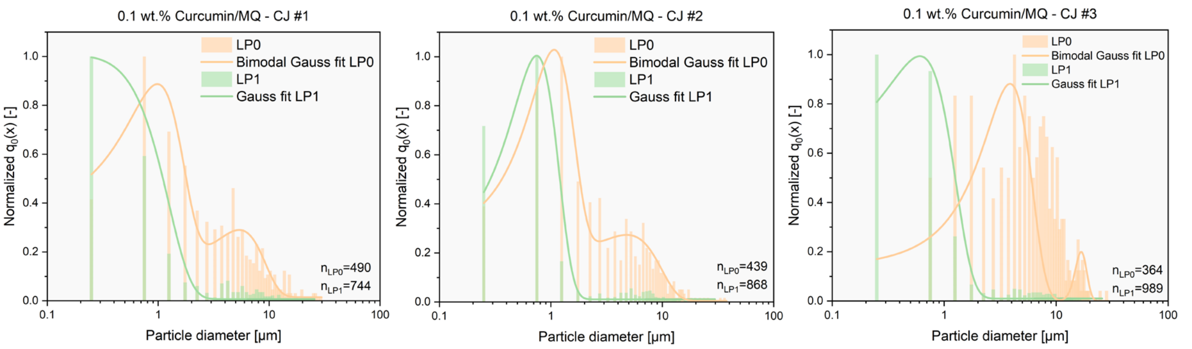

Figure S5: Particle size reduction of 0.1 wt % curcumin in the CJ at 532 nm after LP1 (LFL), three independent determinations via SEM and ImageJ.

Table S3: Modal values of the curcumin particle size distribution at LP0 (untreated) and LP1 (LFL) in the CJ (determined via SEM and Image J).

	**LP0**			
Concentration [wt %]	Modal value 1 [µm]	SD	Modal value 2 [µm]	SD
0.01	1.54	0.29	4.77	0.15
0.05	1.02	0.10	5.42	0.40
0.10	1.04	0.05	5.06	0.29
	**LP1**			
0.01	0.20	0.07	-	-
0.05	0.80	0.08	-	-
0.10	0.70	0.07	-	-

Table S4: Modal values of the CBD particle size distribution at LP0 (untreated) and LP1 (LFL) in the CJ (determined via analytical centrifuge).

	**LP0**					
Concentration [wt %]	Modal value 1 [µm]	SD	Modal value 2 [µm]	SD	Modal value 3 [µm]	SD
0.1	3.56	0.43	9.08	0.37	-	-
0.5	3.58	0.14	9.63	0.30	-	-
1.0	4.06	0.45	9.78	0.10	-	-
	**LP1**					
0.1	3.28	0.12	7.77	0.14	9.36	0.03
0.5	3.35	0.26	6.60	0.88	-	-
1.0	3.88	0.12	5.40	0.10	-	-

➔ all particle size distributions (LP0 and LP1) show additional particles > 10 µm.

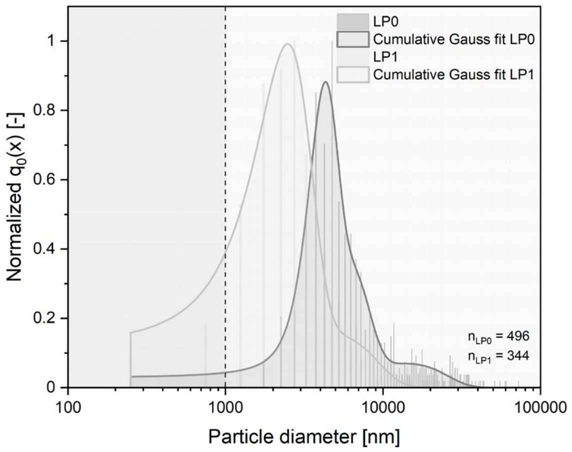

Figure S6: Number-weighted particle size distribution of untreated (LP0) and laser-irradiated (LP1) CBD, determined via SEM and ImageJ.

Chromatograms of the untreated and laser-irradiated model substances.

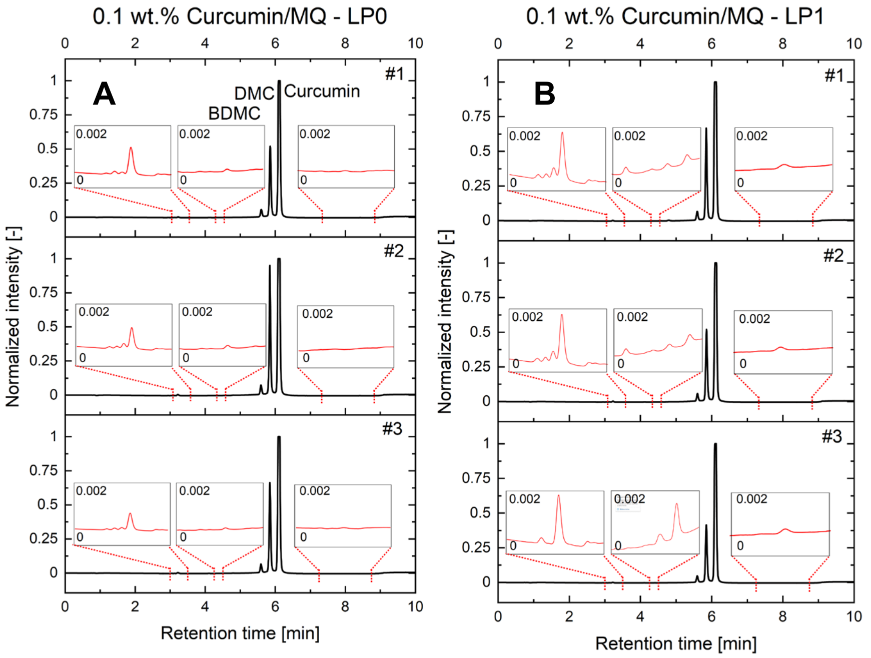

Figure S7: HPLC of 0.1 wt % curcumin for LP0 (untreated) (A) and LP1 (LFL) (B) with the three typical peaks bisdemethoxycurcumin (BDMC), demethoxycurcumin (DMC), and curcumin and additionally appearing degradation products.

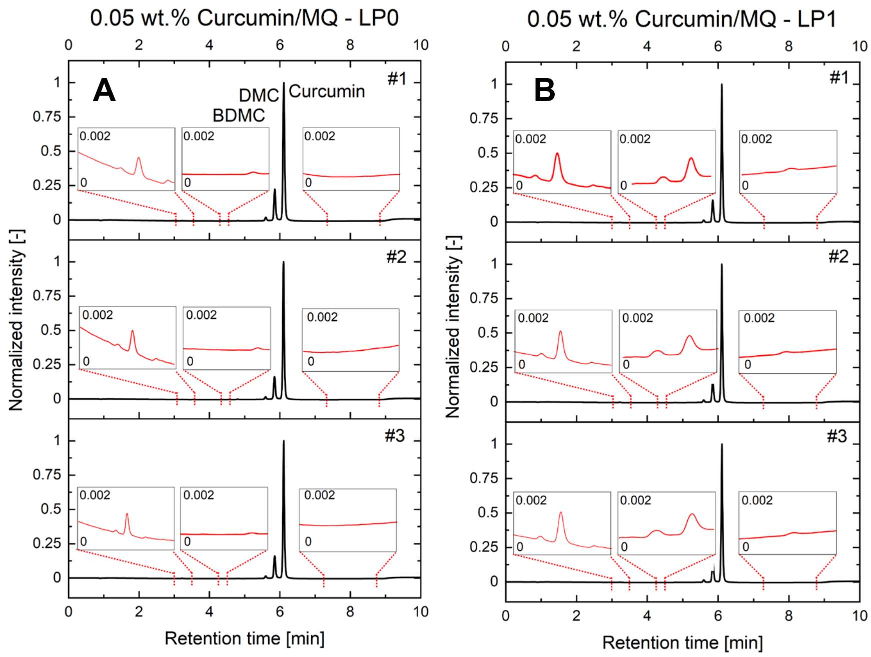

Figure S8: HPLC of 0.05 wt % curcumin for LP0 (untreated) (A) and LP1 (LFL) (B) with the three typical peaks bisdemethoxycurcumin (BDMC), demethoxycurcumin (DMC), and curcumin and additionally appearing degradation products.

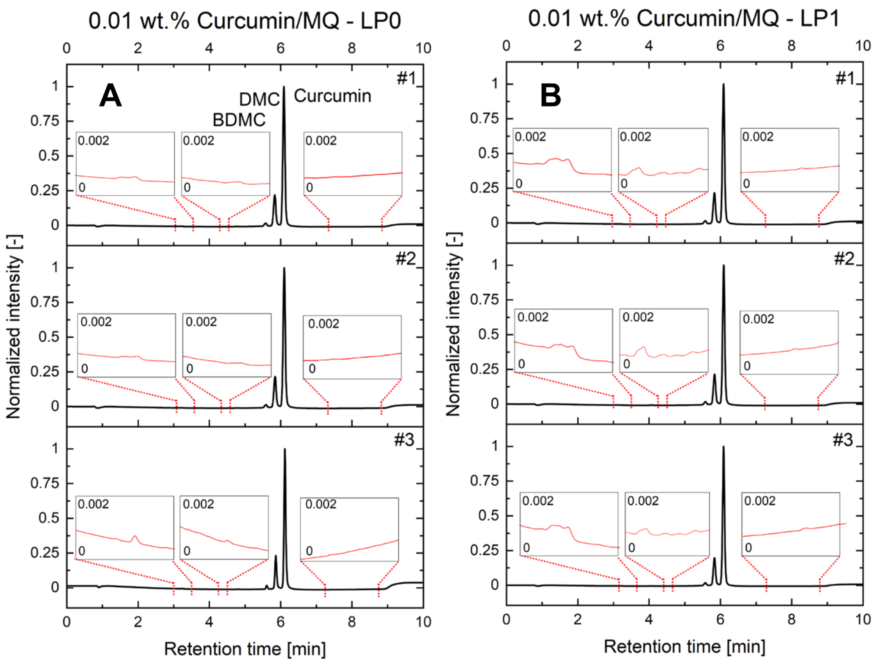

Figure S9: HPLC of 0.01 wt % curcumin for LP0 (untreated) (A) and LP1 (LFL) (B) with the three typical peaks bisdemethoxycurcumin (BDMC), demethoxycurcumin (DMC), and curcumin and additionally appearing degradation products.

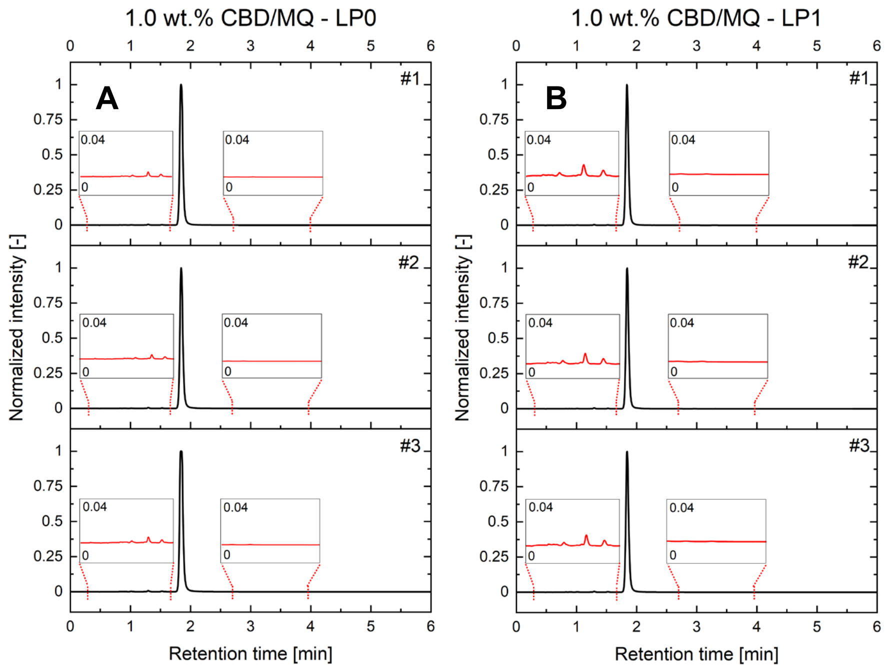

Figure S10: HPLC of 1.0 wt % CBD for LP0 (untreated) (A) and LP1 (LFL) (B) with the typical peak and additionally appearing degradation products.

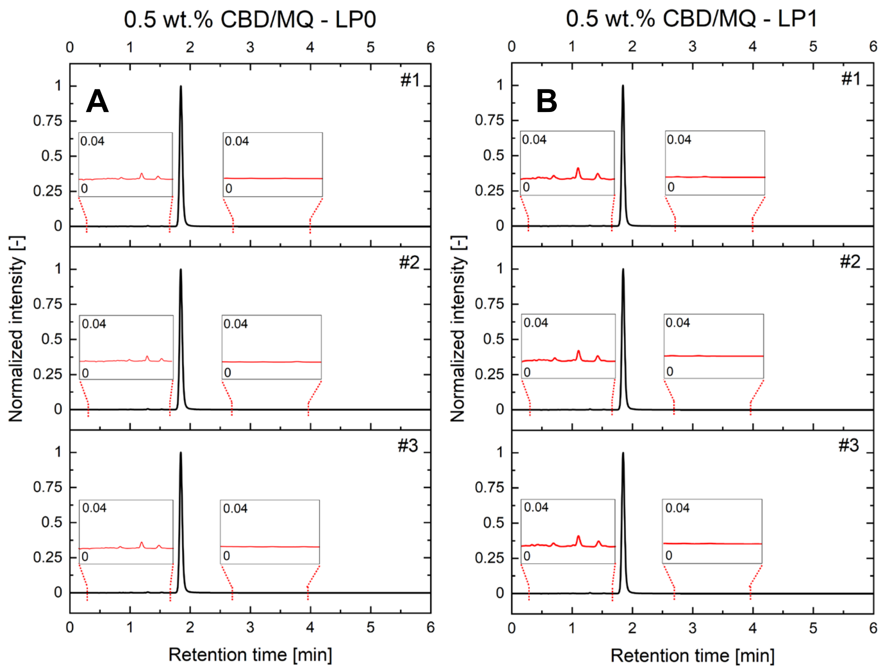

Figure S11: HPLC of 0.5 wt % CBD for LP0 (untreated) (A) and LP1 (LFL) (B) with the typical peak and additionally appearing degradation products.

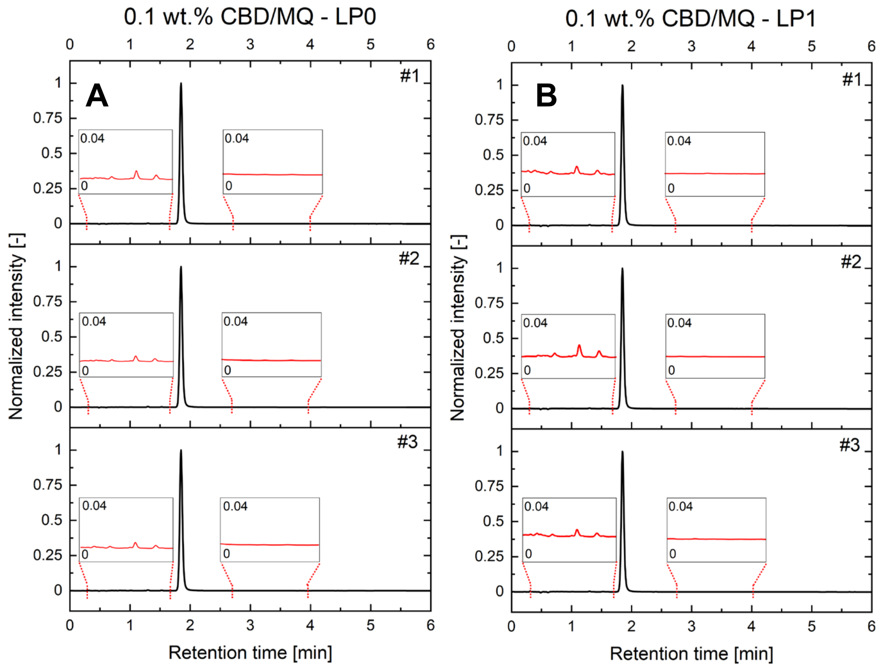

Figure S12: HPLC of 0.1 wt % CBD for LP0 (untreated) (A) and LP1 (LFL) (B) with the typical peak and additionally appearing degradation products.

Table S5: Specific degradation per generated surface for curcumin.

Conc. [wt %]	Conc. [µmol]	**Total degradation** [nmol]	**Generated surface** [cm^2]	**Degradation/generated surface** [nmol cm^{-2}]	SD
0.01	8.14	136.95	6.20	22.09	47.95
0.05	40.72	131.60	5.85	22.48	10.55
0.1	81.44	11.24	14.70	0.76	0.28

Table S6: Specific degradation per generated surface for CBD.

Conc. [wt %]	Conc. [µmol]	**Total degradation** [nmol]	**Generated surface** [cm^2]	**Degradation/generated surface** [nmol cm^{-2}]	SD
0.1	95.40	429.29	115.81	3.71	0.17

Extinction (=absorbance), absorption and scattering values of curcumin and CBD.

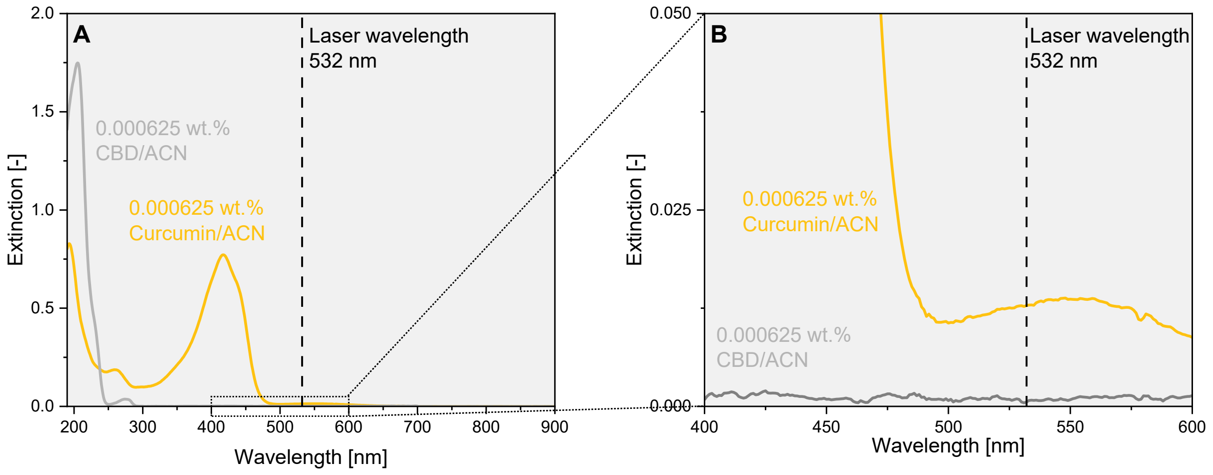

Figure S13: UV–vis extinction spectra of curcumin and CBD dissolved in acetonitrile; full spectra ranging from 190 to 900 nm (A) and zoom ranging from 400 to 600 nm (B).

Table S7: Determination of absorptance, reflectance, and transmittance of curcumin by double integrating sphere at RT and a laser wavelength of 1064 nm. The standard deviation (SD) is based on five laser-powder interactions.

**Layer height** [µm]	**Transmittance** Trans [%]	SD	**Reflectance** Sca [%]	SD	**Absorptance** Abs [%]	SD
250	3.25	0.82	66.39	0.49	30.36	0.95
270	3.69	0.82	66.17	0.49	30.13	0.95
320	3.76	0.82	67.20	0.49	29.04	0.95
330	1.73	0.82	66.59	0.49	31.68	0.95
380	2.24	0.82	67.80	0.49	29.96	0.95
**Mean**	**2.94**	**0.81**	**66.83**	**0.59**	**30.23**	**0.85**

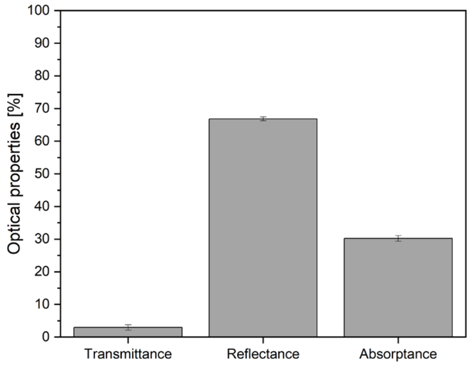

Figure S14: Ratio of transmittance, reflectance, and absorptance of curcumin.

Calculation of the extinction (=absorbance), absorption coefficient and optical penetration depth.

$$\varepsilon_{\text{ext}} = \frac{\log(\frac{I_0}{I})}{cd} \quad \text{(S8)}$$

$$\mu_{\text{abs}} = \text{Abs} * \varepsilon_{ext} * c \quad \text{(S9)}$$

$$d_{\text{opt}} = \frac{1}{\mu_{\text{abs}}} \quad \text{(S10)}$$

Table S8: Parameters measured for extinction (=absorbance) and absorption coefficient calculations curcumin.

Parameter		Value	Unit	Ref.
d	Particle layer in cuvette	7.6	µm	Exp.
I_0	Initial power	25.7.	W	Exp.
I	Measured power	4.5	W	Exp.
c	Concentration	2.7	mmol L^{-1}	Exp.
ε_{ext}	Extinction coefficient	365186	L $(mol\ cm)^{-1}$	Eq. S8
**Abs**	Absorption ratio from extinction	0.31	-	Fig. S14
μ_{abs}	Mean absorption coefficient	309	cm^{-1}	Eq. S9
$\mu_{abs,\ 1\ \mu m}$	Absorption coefficient, 1 µm	258	cm^{-1}	Eq. S9
$\mu_{abs,\ 10\ \mu m}$	Absorption coefficient, 10 µm	377	cm^{-1}	Eq. S9
d_{opt}	Mean opt.penetration depth	32	µm	Eq. S10
$d_{opt,\ 1\ \mu m}$	Opt. penetration depth, 1 µm	39	µm	Eq. S10
$d_{opt,\ 10\ \mu m}$	Opt. penetration depth, 10 µm	27	µm	Eq. S10

Table S9: Parameters measured for extinction (=absorbance) and absorption coefficient calculations CBD.

Parameter		Value	Unit	Ref
d	Particle layer in cuvette	9.8	µm	Exp.
I_0	Initial power	25.7.	W	Exp.
I	Measured power	16.8	W	Exp.
c	Concentration	3.2	mmol L^{-1}	Exp.
ε_{ext}	Extinction coefficient	59853	L $(mol\ cm)^{-1}$	Eq. S8
μ_{abs}	Absorption coefficient	59	cm^{-1}	Eq. S9
d_{opt}	Optical penetration depth	170	µm	Eq. S10

Calculation of the photoacoustic relaxation time.

$$t_{ac} = \frac{d_{opt}}{c_{MP}} \rightarrow \text{if } d_{opt} < a \ (a = \text{particle diameter}) \qquad \text{(S11)}$$

$$t_{ac} = \frac{a}{c_{MP}} \rightarrow \text{if } d_{opt} > a \qquad \text{(S12)}$$

Table S10: Literature and previously calculated values used for the calculation of the photoacoustic relaxation time.

Parameter		Curcumin	CBD	Unit	Ref.
t_p	Pulse duration	10	10	ps	-
c_{MP}	Speed of sound MP	1954	1954	m s^{-1}	[5]
μ_{abs}	Absorption coefficient	309	59	cm^{-1}	Eq. S9
d_{opt}	Optical penetration depth	32	170	µm	Eq. S10
$t_{ac,\ 1\mu m}$	Photoac. relaxation time	512	512	ps	Eq. S12
$t_{ac,\ 10\mu m}$	Photoac. relaxation time	5118	5118	ps	Eq. S12
$t_{ac,\ d\ opt}$	Photoac. relaxation time	17	87	ns	Eq. S11

Calculation of the absorbed fluence. Values in Tables S1 and S8.

$$F_{abs} = \text{Abs} * F_0 \quad \text{(S13)}$$

Calculation of the SMP mass yield and the productivity.

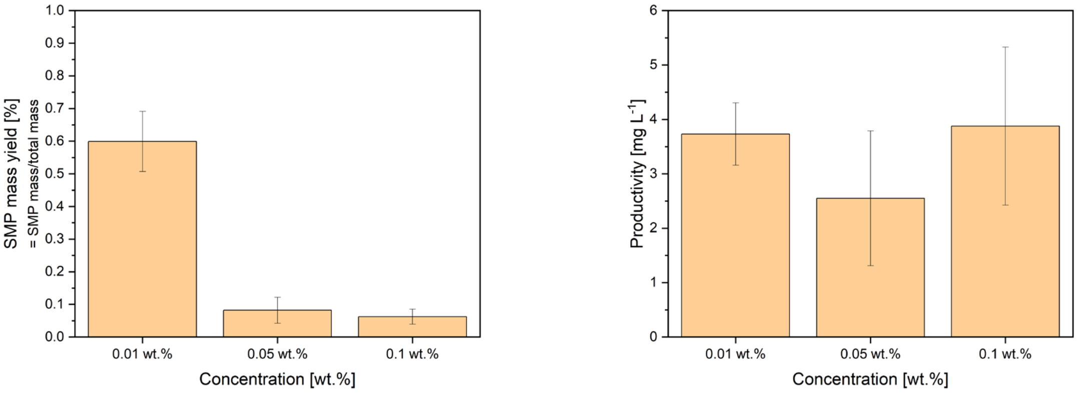

Figure S15: SMP mass yield (= SMP mass/total mass) (A) and productivity of curcumin at different concentrations (B).

Calculation of the temperature increase in curcumin.

For the particle ensemble:[6]

$$\Delta T_e = \frac{\mu_{abs} * F_0}{\rho_{MP} * c_p} \quad \text{(S14)}$$

For individual particles of different sizes:[7]

$$m_p = \rho_{MP} \frac{\pi d^3}{6} \quad \text{(S15)}$$

$$Q_{abs} = \frac{4\sigma_{abs}}{\pi d^2} \quad \text{(S16)}$$

$$\Delta T_i = \frac{\sigma_{abs} * F_0}{m_p * c_p} \quad \text{(S17)}$$

Table S11: Literature values used and calculated temperature increase of curcumin.

Parameter		Value	Unit	Ref.
μ_{abs}	Absorption coefficient	30900	m^{-1}	Eq. S9
F_0	Mean incident fluence	2840	$J\ m^{-2}$	Calc.
ρ_{MP}	Particle density	1310	$kg\ m^{-3}$	Exp.
c_p	Heat capacity curcumin	1931	$J\ (kg\ K)^{-1}$	[8]
T_0	Room temperature	298	K	-
$d_{1\ \mu m}$	Particle diameter	$1*10^{-6}$	m	-
$d_{5\ \mu m}$	Particle diameter	$5*10^{-6}$	m	-
$m_{p,1\ \mu m}$	Particle mass	$6.9*10^{-16}$	kg	Eq. S15
$m_{p,5\ \mu m}$	Particle mass	$8.6*10^{-14}$	kg	Eq. S15
$Q_{abs,\ 1\ \mu m,\ 532\ nm}$	Absorption efficiency	0.16	-	Fig. 6
$Q_{abs,\ 5\ \mu m,\ 532\ nm}$	Absorption efficiency	0.59	-	Fig. 6
$Q_{abs,\ 1\ \mu m,\ 1064\ nm}$	Absorption efficiency	0.08	-	Fig. 6
$Q_{abs,\ 5\ \mu m,\ 1064\ nm}$	Absorption efficiency	0.36	-	Fig. 6
$\sigma_{abs,\ 1\ \mu m,\ 532\ nm}$	Particle absorption efficiency	$1.26*10^{-13}$	m^2	Eq. S16
$\sigma_{abs,\ 5\mu m,\ 532nm}$	Particle absorption efficiency	$1.16*10^{-11}$	m^2	Eq. S16
$\sigma_{abs,\ 1\mu m,\ 1064nm}$	Particle absorption efficiency	$6.28*10^{-14}$	m^2	Eq. S16
$\sigma_{abs,\ 5\mu m,\ 1064nm}$	Particle absorption efficiency	$7.07*10^{-12}$	m^2	Eq. S16
ΔT_e	Temperature incr., ensemble	35	K	Eq. S14
$\Delta T_{i,\ 1\ \mu m,\ 532\ nm}$	Temperature incr., individual	269	K	Eq. S17
$\Delta T_{i,\ 5\ \mu m,\ 532\ nm}$	Temperature incr., individual	199	K	Eq. S17
$\Delta T_{i,\ 1\ \mu m,\ 1064\ nm}$	Temperature incr., individual	135	K	Eq. S17
$\Delta T_{i,\ 5\ \mu m,\ 1064\ nm}$	Temperature incr., individual	121	K	Eq. S17

Calculation of the phase diagram of curcumin (heating-melting-evaporation model [7]).

Table S12: Literature values used for the calculation of the phase diagrams of curcumin.

Parameter		Value	Unit	Ref.
ρ_{MP}	Density	1310	$kg\ m^{-3}$	Exp.
$c_{p,\ solid} = c_{p,\ liquid}$	Heat capacity	1931	$J\ (kg\ K)^{-1}$	[8]
ΔH_m	Melting enthalpy	$1.16*10^{-4}$	$J\ g^{-1}$	[9]
T	Starting temperature	273	K	-
T_0	Initial particle temperature	298	K	-
T_m	Melting temperature	456	K	[9,10]
T_b	Boiling temperature	865	K	[11]

Particle absorption efficiency σ_{abs} (Q_{abs} from MiePlot Figure 7):

$$\sigma_{abs} = \frac{Q_{abs}\pi d^2}{4} \quad \text{(S18)}$$

Particle heating (gray curve in Figure S16):

$$F_0 = \frac{\rho_{MP}*\pi*d^3*c_p*(T-T_0)}{6*\sigma_{abs}} \quad \text{(S19)}$$

Particle melting (red curve in Figure S16):

$$F_0 = \frac{\rho_{MP} * \pi * d^3}{6 * \sigma_{abs}} [c_{p,\,solid} * (T_m - T_0) + \Delta H_m] \quad (S20)$$

Particle evaporation (blue curve in Figure S16):

$$F_0 = \frac{\rho_{MP} * \pi * d^3}{6 * \sigma_{abs}} [c_{p,\,solid} * (T_m - T_0) + \Delta H_m + c_{p,\,liquid} * (T_b - T_m)] \quad (S21)$$

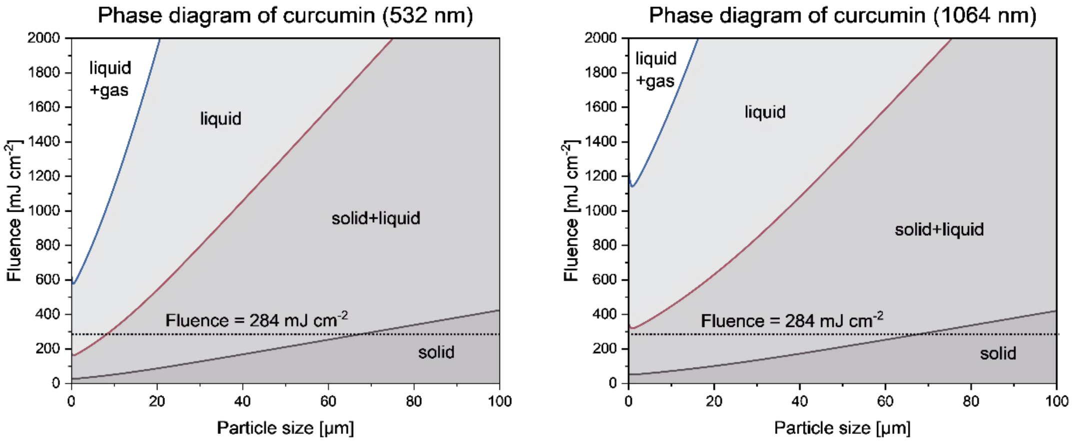

Figure S16: Phase diagram of curcumin for the wavelength of 532 nm (A) and 1064 nm (B) using the heating-melting-evaporation model (Equations S19–S21). The dotted line depicts the used laser fluence of 284 mJ cm^{-2}.

References

1. Huber, M. L.; Perkins, R. A.; Laesecke, A.; Friend, D. G.; Sengers, J. V.; Assael, M. J.; Metaxa, I. N.; Vogel, E.; Mareš, R.; Miyagawa, K. *Journal of Physical and Chemical Reference Data* **2009,** *38* (2), 101–125. doi:10.1063/1.3088050
2. Massalha, T.; Digilov, R. M. *American Journal of Physics* **2013,** *81* (10), 733–737. doi:10.1119/1.4819196
3. Mi, J.; Xu, M.; Zhou, T. *Physics of Fluids* **2013,** *25* (7). doi:10.1063/1.4811403
4. Xia, Q.; Lei, S.; Ma, J.; Zhong, S. *International Journal of Heat and Fluid Flow* **2014,** *50*, 456–466. doi:10.1016/j.ijheatfluidflow.2014.10.019

5. Adhikari, K.; Flurchick, K. M.; Valenzano, L. *Chemical Physics Letters* **2015,** *630*, 44–50. doi:10.1016/j.cplett.2015.04.024
6. Paltauf, G.; Schmidt-Kloiber, H. *Appl. Phys. A* **1999** (68), 525–531. doi:10.1007/s003399900033
7. Pyatenko, A.; Wang, H.; Koshizaki, N.; Tsuji, T. *Laser & Photonics Reviews* **2013,** *7* (4), 596–604. doi:10.1002/lpor.201300013
8. G. Jeevarathinam and T. Pandiarajan. *Advances in Life Sciences* **2016,** *5* (12), 5167–5170.
9. Cui, Z.; Yao, L.; Ye, J.; Wang, Z.; Hu, Y. *Journal of Molecular Liquids* **2021,** *338*, 116795. doi:10.1016/j.molliq.2021.116795
10. Ravindranath, V.; Chandrasekhara, N. *Toxicology* **1981,** *20* (2-3), 251–257. doi:10.1016/0300-483x(81)90056-1
11. Muerth, H. Verwendung des Verfahrens und der Vorichtungen zum Verdampfen von Curcumin-Pulver mit einem Hochleistungs-Verdampfer zwecks Erzeugen eines inhalierbaren Curcumin-Aerosols. 10 2015 008 823.8, Jul 15, 2015.
